# Extracorporeal carbon dioxide removal for patients with acute respiratory failure: a systematic review and meta-analysis

**DOI:** 10.1080/07853890.2023.2172606

**Published:** 2023-03-01

**Authors:** Zhifeng Zhou, Zhengyan Li, Chen Liu, Fang Wang, Ling Zhang, Ping Fu

**Affiliations:** a Division of Nephrology, Kidney Research Institute, West China Hospital of Sichuan University, Chengdu, China; b State Key Laboratory of Kidney Diseases, National Clinical Research Center for Kidney Diseases, First Medical Center of Chinese, PLA General Hospital, Beijing, China; c Division of Radiology, West China Hospital of Sichuan University, Chengdu, China

**Keywords:** Extracorporeal carbon dioxide removal, acute respiratory failure, mortality, meta-analysis

## Abstract

**Background:**

Acute respiratory failure (ARF) is a common clinical critical syndrome with substantial mortality. Extracorporeal carbon dioxide removal (ECCO_2_R) has been proposed for the treatment of ARF. However, whether ECCO_2_R could provide a survival advantage for patients with ARF is still controversial.

**Methods:**

Electronic databases (PubMed, Embase, Web of Science, and the Cochrane database) were searched from inception to 30 April 2022. Randomized controlled trials (RCTs) and observational studies that examined the following outcomes were included: mortality, length of hospital and ICU stay, intubation and tracheotomy rate, mechanical ventilation days, ventilator-free days (VFDs), respiratory parameters, and reported adverse events.

**Results:**

Four RCTs and five observational studies including 1173 participants with ARF due to COPD or ARDS were included in this meta-analysis. Pooled analyses of related studies showed no significant difference in overall mortality between ECCO_2_R and control group, neither in RCTs targeted ARDS or acute hypoxic respiratory failure patients (RR 1.05, 95% CI 0.83 to 1.32, *p* = 0.70, I^2^ =0.0%), nor in studies targeted patients with ARF secondary to COPD (RR 0.80, 95% CI 0.58 to 1.11, *p* = 0.19, I^2^ =0.0%). A shorter duration of ICU stay in the ECCO_2_R group was only obtained in observational studies (WMD −4.25, *p* < 0.01), and ECCO_2_R was associated with a longer length of hospital stay (*p* = 0.02). ECCO_2_R was associated with lower intubation rate (*p* < 0.01) and tracheotomy rate (*p* = 0.01), and shorter mechanical ventilation days (*p* < 0.01) in comparison to control group in ARF patients with COPD. In addition, an improvement in pH (*p* = 0.01), PaO2 (*p* = 0.01), respiratory rate (*p* < 0.01), and PaCO2 (*p* = 0.04) was also observed in patients with COPD exacerbations by ECCO_2_R therapy. However, the ECCO_2_R-related complication rate was high in six of the included studies.

**Conclusions:**

Our findings from both RCTs and observational studies did not confirm a significant beneficial effect of ECCO_2_R therapy on mortality. A shorter length of ICU stay in the ECCO_2_R group was only obtained in observational studies, and ECCO_2_R was associated with a longer length of hospital stay. ECCO_2_R was associated with lower intubation rate and tracheotomy rate, and shorter mechanical ventilation days in ARF patients with COPD. And an improvement in pH, PaO2, respiratory rate and PaCO2 was observed in the ECCO_2_R group. However, outcomes largely relied on data from observational studies targeted patients with ARF secondary to COPD, thus further larger high-quality RCTs are desirable to strengthen the evidence on the efficacy and benefits of ECCO_2_R for patients with ARF.

**Key messages**
1. ECCO_2_R therapy did not confirm a significant beneficial effect on mortality.
2. ECCO_2_R was associated with lower intubation and tracheotomy rate, and shorter mechanical ventilation days in patients with ARF secondary to COPD.
3. An improvement in pH, PaO2, respiratory rate, and PaCO2 was observed in ECCO_2_R group in patients with COPD exacerbations.
4. Evidence for the future application of ECCO_2_R therapy for patients with ARF. The protocol of this meta-analysis was registered on PROSPERO (CRD42022295174).

## 1. Introduction

Acute respiratory failure (ARF) is a common clinical critical syndrome, which has a substantial mortality due to damage to the function of multiple organs, such as the heart, brain, and kidney. Acute respiratory distress syndrome (ARDS) and chronic obstructive pulmonary disease (COPD) are two major causes of ARF [1,2]. Currently, non-invasive ventilation (NIV) is a common therapy for the treatment of ARF. However, a great number of these patients will fail NIV and require invasive mechanical ventilation (IMV) [3], and evidence has shown that IMV with high tidal volume or high ventilation pressure can cause lung injury and increase mortality [4]. Low tidal volumes and limited plateau pressures have been confirmed to provide a survival advantage [5]. However, with concerns of progressive hypercapnia and potential adverse physiological consequences, low tidal volume ventilation is difficult to implement [6]. To solve this problem, a technique of artificial respiratory support called extracorporeal carbon dioxide removal (ECCO_2_R) that combines MV with extracorporeal life support has emerged.

Developed from the principle of extracorporeal membrane oxygenation (ECMO) systems, ECCO_2_R can provide carbon dioxide removal with lower tidal volumes to reduce lung injury and provide respiratory support for recovery. This technology was first described by Gattinoni in the late 1970s [7,8], but its wide clinical use is limited due to demanding technical requirements and major complications [9]. Recently, modern developments in ECCO_2_R technology and the emergence of integrated ECCO_2_R systems with higher CO_2_ removal efficiency have stimulated renewed interest [10,11]. Currently, an increasing number of studies have provided evidence for the role of the ECCO_2_R system in the treatment of patients with ARF. When extremely abnormal hypoxemia or extremely abnormal hypercapnia exists, we could consider treatment with ECCO_2_R. And when improvement had not been observed after a few days of lung protective ventilation, treatment with ECCO_2_R could also be considered [12]. However, the efficacy and benefits of ECCO_2_R for patients with ARF are still controversial. Therefore, in order to define the current understanding of ECCO_2_R in patients with ARF, we undertook a meta-analysis to assess the potential efficacy of ECCO_2_R.

## 2. Methods

We conducted a meta-analysis in accordance with the Preferred Reporting Items for Systematic Reviews and Meta-analyses (PRISMA) guidelines [13], and the protocol of this meta-analysis was registered on PROSPERO (CRD42022295174).

### 2.1. Search strategy

We systematically searched all relevant publications in PubMed, Embase, Web of Science and the Cochrane database from inception to 30 Apr 2022. The search terms were as follows: ‘extracorporeal carbon dioxide removal’ or ‘ECCO_2_R’ or ‘extracorporeal lung assist’ or ‘interventional lung assist’ and ‘acute respiratory failure’ or ‘acute respiratory distress syndrome’ or ‘chronic obstructive pulmonary disease’ (Supplementary File 1). The search process was performed and confirmed by two independent reviewers. They screened the retrieved titles and abstracts to exclude irrelevant studies and performed a full-text review when it was difficult to estimate whether a study should be included.

### 2.2. Selection criteria

Participants: Adult patients with ARF.

Interventions: Arteriovenous extracorporeal carbon dioxide removal (AV-ECCO_2_R) or veno-venous extracorporeal carbon dioxide removal (VV-ECCO_2_R) device. The intervention of the control group was the traditional therapy mentioned in the included studies.

Types of outcome measures: Mortality (hospital, ICU or 28-day mortality), length of hospital and ICU stay, intubation and tracheotomy rate, mechanical ventilation days, ventilator-free days (VFDs), respiratory parameters (pH, PaO2, PaCO2, PaO2/FiO2 or respiratory rate), reported adverse events (major or minor adverse events), and cost.

Type of studies: RCTs and observational studies concerning ECCO_2_R versus traditional therapies for adult patients with ARF.

### 2.3. Data extraction

The inclusion criteria were as follows: (1) studies that enrolled adult patients diagnosed with ARF who had been treated with ECCO_2_R versus traditional therapies; (2) RCTs or observational studies (for example, case–control or cohort studies); (3) studies reporting the following outcomes: mortality, length of hospital and ICU stay, intubation and tracheotomy rate, mechanical ventilation days, VFDs, respiratory parameters, reported adverse events, and cost; and (4) sufficient data available to calculate risk ratios (RRs), weighted mean differences (WMDs) or standardized mean differences (SMDs) with 95% confidence intervals (CIs).

Exclusion criteria: (1) no relevant data; (2) studies without a control group; (3) nonhuman studies; and (4) only a meeting paper or abstract was published without the full text.

Two investigators independently extracted data from the included studies, and disagreements were resolved by a third author. The following data were extracted: (1) study characteristics: publication year, author names, study design, target population, number of participants, age, and sex; (2) study intervention and control, type of ECCO_2_R device; and (3) outcome characteristics: mortality, length of hospital and ICU stay, intubation and tracheotomy rate, mechanical ventilation days, VFDs, respiratory parameters, reported major or minor adverse events, and cost.

### 2.4. Quality assessment

Two investigators independently evaluated the quality of the included studies, and if they could not reach consensus, disagreements would be settled by discussion with a third reviewer until consensus was reached. The Cochrane risk of bias tool [14] and the Newcastle–Ottawa Scale (NOS) [15] were used to assess the quality of RCTs and observational studies, respectively. For each RCT, selection bias (random sequence generation and allocation concealment), performance bias (blinding of participants and personnel), detection bias (blinding of outcome), attrition bias (incomplete outcome data), reporting bias (selective reporting) and other biases were categorized as ‘low’, ‘unclear’ or ‘high’ risk of bias. For observational studies, we adopted the NOS, which focused on three categories: selection, comparability, and outcome. And a judgment of a ‘low’ risk of bias was scored 1, and an ‘unclear’ or ‘high’ risk of bias was scored 0. Studies were considered high quality if they scored above 5 points.

### 2.5. Statistical analysis

Data analyses were performed using Stata SE, version 14 (StataCorp, College Station, TX, USA). WMDs or SMDs with 95% CIs were calculated for continuous variables, and RRs with 95% CIs were calculated for dichotomous variables. Heterogeneity was evaluated using the I^2^ statistic. I^2^ values of 0–24.9%, 25–49.9%, 50–74.9%, and 75–100% represented no, low, moderate, and significant heterogeneity, respectively [16,17]. When the homogeneity across studies was insufficient, a random effects model would be performed. Subgroup analyses were performed to evaluate the possible explanations of heterogeneity. *p* < 0.05 was considered statistically significant. In addition, Begg’s funnel plots and Egger’s test were also performed to analyze potential publication bias.

## 3. Results

### 3.1. Search results and characteristics of the included studies

**Figure 1.**
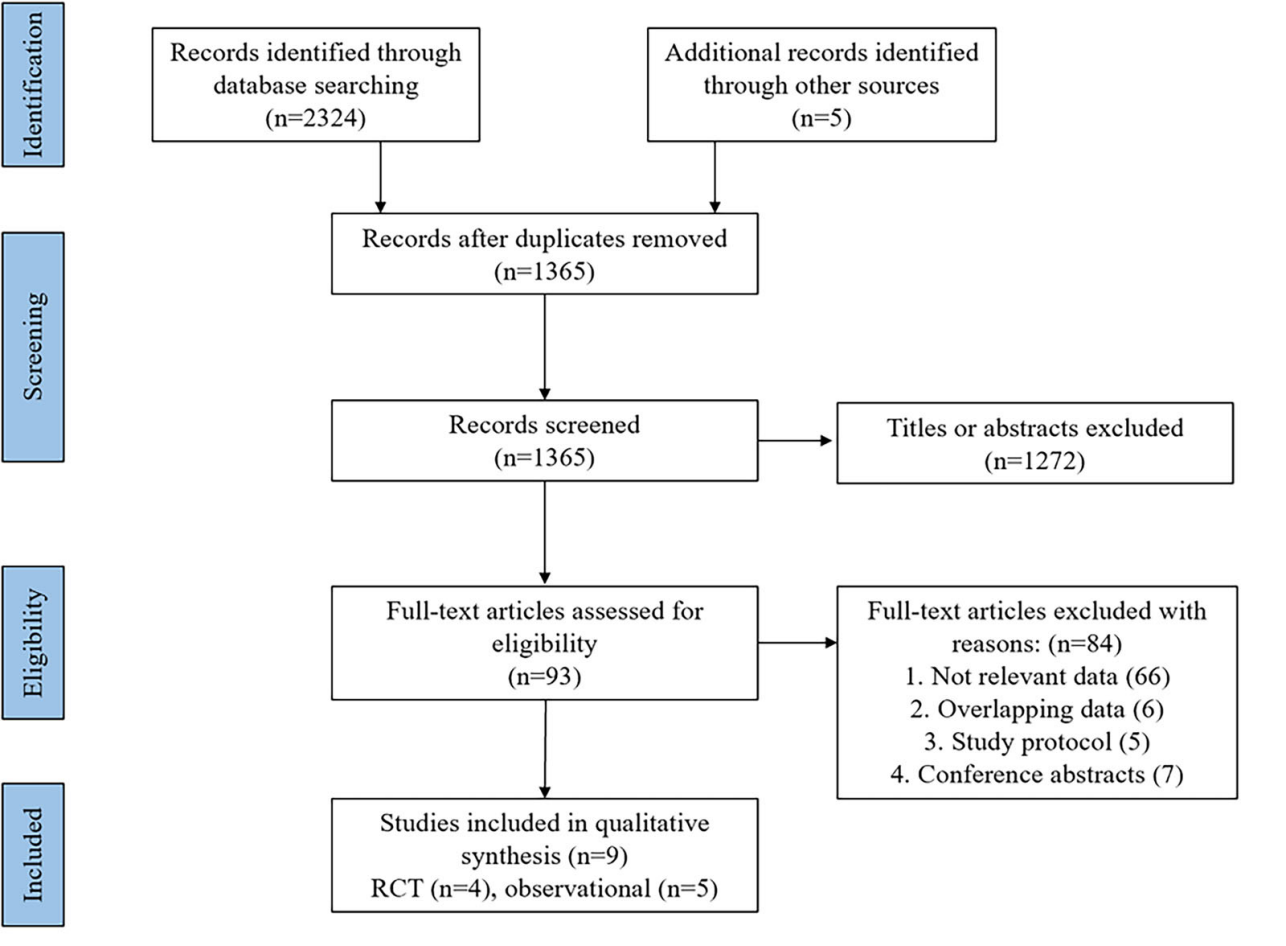
Flow chart of study selection.

A flowchart of the study selection process is presented in Figure 1. A total of 2329 articles were identified from our initial search, of which 964 articles were excluded because of deduplication. After evaluating the title and abstract of each article, we excluded 1272 articles that did not meet the inclusion criteria. Subsequently, 93 articles were read for full text for further evaluation, and 84 articles were excluded for the following reasons: irrelevant data (*n* = 66), overlapping data (*n* = 6), study protocol (*n* = 5), and conference abstracts (*n* = 7). Finally, 4 RCTs [18–21] and five observational studies [22–26] were included in this meta-analysis. The eligible studies were conducted from 1994 to 2022 with a total number of 1173 patients. Among the included studies, two were conducted in multiple countries [18,23], two were from the United Kingdom [19,21], and other studies were conducted in France [22], America [20], Turkey [26], Germany [25], Italy [24]. The sample sizes ranged from 18 to 412, with a median of 50 patients. A variety of outcomes were recorded in these studies, including mortality, length of stay (days), intubation rate, tracheotomy rate, mechanical ventilation support, VFDs, respiratory parameters, and adverse events. The baseline characteristics and outcomes of the RCTs and observational studies fulfilling the inclusion criteria are shown in Tables 1 and 2. And the inclusion criteria and exclusion criteria of participants in the included studies are shown in Supplementary Table 1.

**Table 1.** Basic characteristics of included studies.

Studies	Country	**Study** **design**	**Target** **population/setting**	**Patients** **(N)**	**Males** **(%)**	Age (year)	PaO2/FIO2 ratio	PaCO2 (mmHg)	Intervention	Control
Bein et al. [18]	Germany and Austria	RCT	Patients with ARF secondary to severe ARDS/ICU	I: 40 C: 39	I: 95 C: 77	I: 49.8 ± 12 C: 48.7 ± 17	<200 I: 152 ± 37 C: 168 ± 37	I: 57.3 ± 12 C: 54.3 ± 9	Low tidal volumes mechanical ventilation (3 ml/kg) with AV-ECCO_2_R	Mechanical ventilation using conventional tidal volumes (6 ml/kg)
McNamee et al. [19]	UK	RCT	Patients receiving iMV for ARF/ICU	I: 202 C: 210	I: 68 C: 62	I: 60 (51, 69) C: 62 (50, 70)	<150 I: 118.1 (96.0, 134.3) C: 115.5 (93.8, 132.8)	I: 53.8 (47.3, 62.7) C: 54.6 (48.0, 62.3)	Low tidal volumes mechanical ventilation with VV-ECCO_2_R	Lung protective ventilation alone
Morris et al. [20]	America	RCT	Patients with ARF secondary to severe ARDS/ICU	I: 21 C: 19	I: 38 C: 47	I: 33 ± 3.1 C: 38 ± 3.3	I: 62.6 ± 4.2 C: 63.8 ± 3.8	I: 50 ± 3 C: 48 ± 4	LFPPV + VV- ECCO_2_R	Standardized CPPV
Barrett et al. [21]	UK	RCT	Patients presenting with ARF due to AECOPD/ICU	I: 9 C: 9	I: 56 C: 33	I: 65 (63, 71) C: 69 (61, 71)	/	I: 73.1 (61.1, 73.4) C:68.9 (67.1, 77.3)	VV-ECCO2R added to NIV	Standard therapy using NIV
Azzi et al. [22]	France	Observational	Patients with consecutive AECOPD who experienced NIV failure/ICU	I: 26 C: 25	I: 77 C: 68	I: 67 ± 12 C: 72 ± 11	/	I: 86 ± 21 C: 82 ± 24	VV-ECCO_2_R	iMV after failing NIV
Braune et al. [23]	Germany, Austria, Netherlands	Observational	Patients with ARF secondary to COPD refractory to NIV/ICU	I: 25 C: 25	I: 48 C: 52	I: 67 (51, 83) C: 69 (55, 82)	I: 209.3 (106.2, 476.0) C: 201.0 (58.0, 466.0)	I: 81.5 (53.8, 126) C: 79.5 (48.4, 117)	Pump-driven, VV-ECCO_2_R device	iMV after failing NIV
Del Sorbo et al. [24]	Italy	Observational	Patients treated with NIV for ARF due to AECOPD/ICU	I: 25 C: 21	/	I: 70.7 ± 7.1 C: 70.4 ± 9.8	I: 211 (138, 248) C: 187 (141, 245)	I: 74 (64, 89) C: 78 (70, 88)	NIV-Plus VV-ECCO_2_R	NIV-Only
Kluge et al. [25]	Germany	Observational	Patients with ARF secondary to COPD unresponsive to NIV/ICU	I: 21 C: 21	I: 48 C: 43	I: 58 (27, 80) C: 58 (23, 79)	I: 208 (153, 396) C: 179 (113, 591)	I: 84.0 (54.2, 131) C: 65.0 (39.2, 108)	AV-ECCO_2_R therapy by means of a PECLA device	iMV after failing NIV
İnal et al. [26]	Turkey	Observational	Patients with ARF secondary to COPD or ARDS (data about COPD is collected)/ICU	I: 49 C: 207	I: 45 C: 45	I: 68 (51, 93) C: 69 (41, 97)	I: 129 ± 13 C: 133 ± 14	I: 78 ± 21 C: 75 ± 18	VV-ECCO_2_R therapy in addition to conventional treatments	Conventional treatments alone

iMV: invasive mechanical ventilation; ARF: acute respiratory failure; COPD: chronic obstructive pulmonary disease; ARDS: acute respiratory distress syndrome; ICU: intensive care unit; NIV: noninvasive ventilation; ECCO_2_R: extracorporeal carbon dioxide removal; PECLA: pumpless extracorporeal lung-assist.

**Table 2.** Clinical outcomes and complications of included studies.

Studies	Clinical outcomes				Adverse events
	Mortality	Length of stay (days)	Intubation/extubation	Respiratory parameters	
Bein et al. [18]	Hospital: 7/40 (18%) vs 6/39 (15%)	ICU: 31.3 ± 23 vs 22.9 ± 11; hospital: 46.7 ± 33 vs 35.1 ± 17	VFD-28 (days): 10.0 ± 8 vs 9.3 ± 9; VFD-60 (days): 33.2 ± 20 vs 29.2 ± 21; non-pulmonary organ failure free days-60 (days): 21.0 ± 14 vs 23.9 ± 15	/	AV-ECCO_2_R related adverse events: 3/40
McNamee et al. [19]	28-day: 76/200 (38%) vs 74/207 (36%); 90-day: 83/200 (42%) vs 81/205 (40%)	ICU: 14 (7, 26) vs 13 (7, 22); hospital: 22 (8, 39) vs 18 (9, 35)	VFD-28 (days): 7.1 ± 8.8 vs 9.2 ± 9.3;	pH: 7.30 (7.25, 7.37) to 7.31 ± 0.09 vs 7.30 (7.24, 7.37) to 7.31 ± 0.1; PaCO2 (mmHg): 53.8 (47.3, 62.7) to 55.8 ± 14.4 vs 54.6 (48, 62.3) to 56.6 ± 13.8; P/F ratio: 118.1 (96, 134.3) to 153.1 ± 84 vs 115.5 (93.8, 132.8) to 145.1 ± 48.3; RR: 24 (20, 28) to 23.9 ± 5.02 vs 24 (20, 28) to 23.9 ± 5.24	Adverse events: 106/202 vs 48/210; major adverse events: 62/202 vs 18/210
Morris et al. [20]	28-day: 14/21 (67%) vs 11/19 (58%)	ICU: 23.8 ± 18.3 vs 24.2 ± 19.2; hospital: 26.9 ± 22.5 vs 28.8 ± 24.8	CPPV duration (days): 4.46 ± 10.1 vs 19.3 ± 16.1; ECCO_2_R duration (days): 8.7 ± 1.7	/	Number of patients with major complications (not number of episodes): 34 vs 16 Major bleeding: 17/21 vs 1/19;
Barrett et al. [21]	Hospital: 3/9 (33%) vs 1/9 (11%); ICU: 3/9 (335) vs 0/9 (0%); 90-day: 4/9 (44%) vs 2/9 (22%)	ICU: 6.7 (5.5, 7.3) vs 1.9 (1.7, 2.2); hospital: 10 (9.2, 14.1) vs 5.2 (4.3, 8.9)	NIV duration (days): 0.3 ± 0.1 vs 1.6 ± 1.5	pH: 7.27 (7.25, 7.29) to 7.39 (7.37, 7.42) vs 7.27 (7.21, 7.27) to 7.38 (7.36, 7.40); PaCO2 (kPa): 9.34 (8.49, 10.2) to 8.02 (6.57, 8.3) vs 9.16 (8.23, 10.02) to 7.40 (7.16, 8.08); RR: 29 (26, 32) to 17 (16, 23) vs 24 (20, 28) to 20.5 (20, 22.75)	No severe or life-threatening complications in either group.
Azzi et al. [22]	28-day: 3/26 (12%) vs 4/25 (16%); 90-day: 4/26 (15%) vs 7/25 (28%); ICU: 2/26 (8%) vs 7/25 (28)	ICU: 18 ± 14 vs 30 ± 43; hospital: 29 ± 22 vs 49 ± 53	Intubation rate: 4/26 vs 25/25; tracheotomy: 2/26 vs 5/25; ECCO_2_R duration (days): 5.4 ± 4 vs iMV duration (days): 27 ± 43	pH: 7.24 ± 0.05 to 7.41 ± 0.06 vs 7.23 ± 0.13 to 7.39 ± 0.06; PaCO2 (mmHg): 86 ± 21 to 53 ± 10 vs 82 ± 24 to 52 ± 13;	(ECCO_2_R) Major bleeding: 6/26; minor bleeding: 5/26; (control) ventilator associated pneumonia: 8/25; hemodynamic instability with catecholamine administration requirement: 3/26 vs 16/25
Braune et al. [23]	28-day: 4/25 (16%) vs 3/25 (12%); 90-day: 7/25 (28%) vs 7/25 (28%); hospital: 6/25 (24%) vs 3/25 (12%)	ICU: 28.9 (8.0, 100.0) vs 24.0 (2.0, 66.0); hospital: 36.9 (9.0, 100.0) vs 37.0 (12.0, 248.0)	14/25 avoided intubation vs 0/21 avoided intubation; iMV duration (days): 8.3 (0, 60.0) vs 13.7 (1.0, 52.0); tracheotomy: 9/25 (36%) vs 15/25 (60%)	ECCO_2_R: pH: 7.26 (7.15, 7.33) to 7.42 (7.34, 7.48); PaCO2 (mmHg): 82.0 (68.0, 94.0) to 52.0 (44.0, 66.0); RR: 25 (24, 34) to 20 (15, 23)	Major adverse events: 14 vs 2; minor adverse events: 11 vs 10 Major bleeding: 11/25 vs 2/25; minor bleeding: 10/25 vs 10/25
Del Sorbo et al. [24]	Hospital: 2/25 (8%) vs 8/21 (35%)	ICU: 8 (7, 10) vs 12 (6, 15); hospital: 24 (21, 28) vs 22 (13, 36)	3/25 requiring intubation vs 7/21 requiring intubation	pH: 7.27 (7.25, 7.28) to 7.34 (7.32, 7.39) vs 7.28 (7.23, 7.30) to 7.28 (7.17, 7.30); PaCO2 (mmHg): 88.0 (67.0, 96.0) to 63.0 (52.0, 84.0) vs 82.0 (76.0, 89.0) to 80.0 (66.0, 104.5); P/F ratio: 168 (133, 210) to 178 (131, 203) vs 176 (152, 233) to 235 (212, 262); RR (breaths/min): 32 (29, 35) to 22 (18, 24) vs 30 (28, 32) to 27 (25, 31)	13 patients (52%) experienced adverse events related to ECCO2R: mechanical events (9); patient-related events (4)
Kluge et al. [25] and Braune et al. [42]	28-day: 5/21 (24%) vs 4/21 (19%); 6-month: 7/21 (33%) vs 7/21 (33%)	ICU: 15 (4, 137) vs 30 (4, 66); hospital: 23 (4, 137) vs 42 (4, 248); (COPD) ICU: 11 (4, 23) vs 35 (4, 66); hospital: 17 (4, 43) vs 51 (4, 248);	19/21 avoided intubation vs 0/21 avoided intubation; duration of PECLA support (days): 9 (1, 116), duration of MV (days): 21 (1, 47); tracheotomy: 2/21 (10%) vs 14/21 (67%)	ECCO_2_R: pH: 7.28 (7.10, 7.41) to 7.44 (7.27, 7.56); PaCO2 (mmHg): 84.0 (54.2, 131.0) to 52.1 (33.0, 70.1); PaO2 (mmHg): 84.1 (67.2, 158.4) to 80.0 (57.3, 153.2); P/F ratio: 208 (153, 396) to 209 (134, 570); RR: 27 (15, 36) to 21 (11, 32)	ECCO_2_R: 2 major bleeding and 7 minor bleeding
İnal et al. [26]	28-day: 24/75 (32%) vs 133/320 (42%); ARDS: 7/26 (27%) vs 39/113 (35%); COPD: 17/49 (35%) vs 94/207 (45%)	ICU: 17 (7, 28) vs 26 (5, 45); ARDS: 15 (7, 23) vs 29 (13, 52); COPD: 17 (7, 19) vs 21 (5, 37)	iMV duration (days): 11 (8, 19) vs 14 (7, 22); ARDS: 10 (8, 17) vs 15 (9, 25); COPD: 11 (7, 18) vs 15 (8, 21)	pH: 7.156 ± 0.07 to 7.339 ± 0.05 vs 7.184 ± 0.02 to 7.265 ± 0.09; PaCO2 (mmHg): 81 ± 21 to 53 ± 9 vs 77 ± 19 to 71 ± 16; PaO2 (mmHg): 61 ± 6 to 82 ± 8 vs 59 ± 7 to 71 ± 9; ECCO_2_R: P/F ratio: 89 ± 11 to 150 ± 53	No severe adverse effects related to procedure were mentioned

iMV: invasive mechanic ventilation; COPD: chronic obstructive pulmonary disease; ARDS: acute respiratory distress syndrome; ICU: intensive care unit; ECCO_2_R: extracorporeal carbon dioxide removal; VFD: ventilator-free day; CPPV: conventional positive pressure ventilation.

### 3.2. Quality assessment

For the evaluation of the quality of the included studies, we used the Kappa coefficient to test the consistency, and we showed a substantial coefficient with a Kappa value of 0.90. Disagreements were resolved by a third party through consensus. The details of the risk of bias are summarized in Supplementary File 2. Four RCTs were assessed for quality by the Cochrane risk of bias tool. Two RCT was judged to have a low risk of bias, and another 2 were judged to be at unclear risk of bias. Due to the complex nature of the intervention, neither RCT was double-blinded. However, the authors judged that the primary outcomes (mortality and length of hospital or ICU stay) are not likely to be influenced by lack of blinding. For observational studies, all showed comparatively high quality based on an NOS score ≥ 6, and detail of the NOS quality assessment of the included observational studies is listed in Supplementary File 2.

### 3.3. Mortality

**Figure 2.**
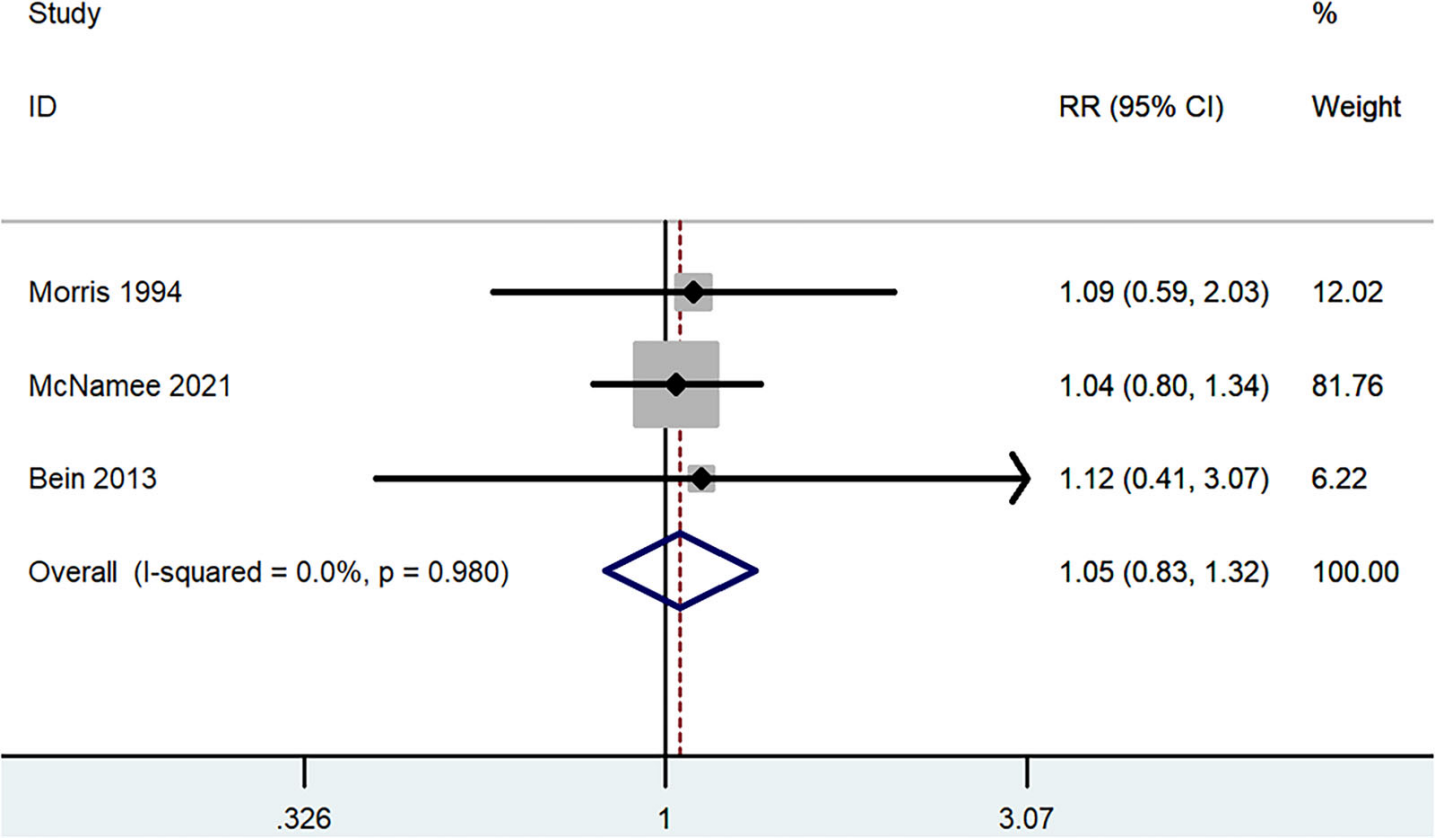
Forest plot for overall mortality within RCTs targeted patients with ARF secondary to ARDS or acute hypoxic respiratory failure.

**Figure 3.**
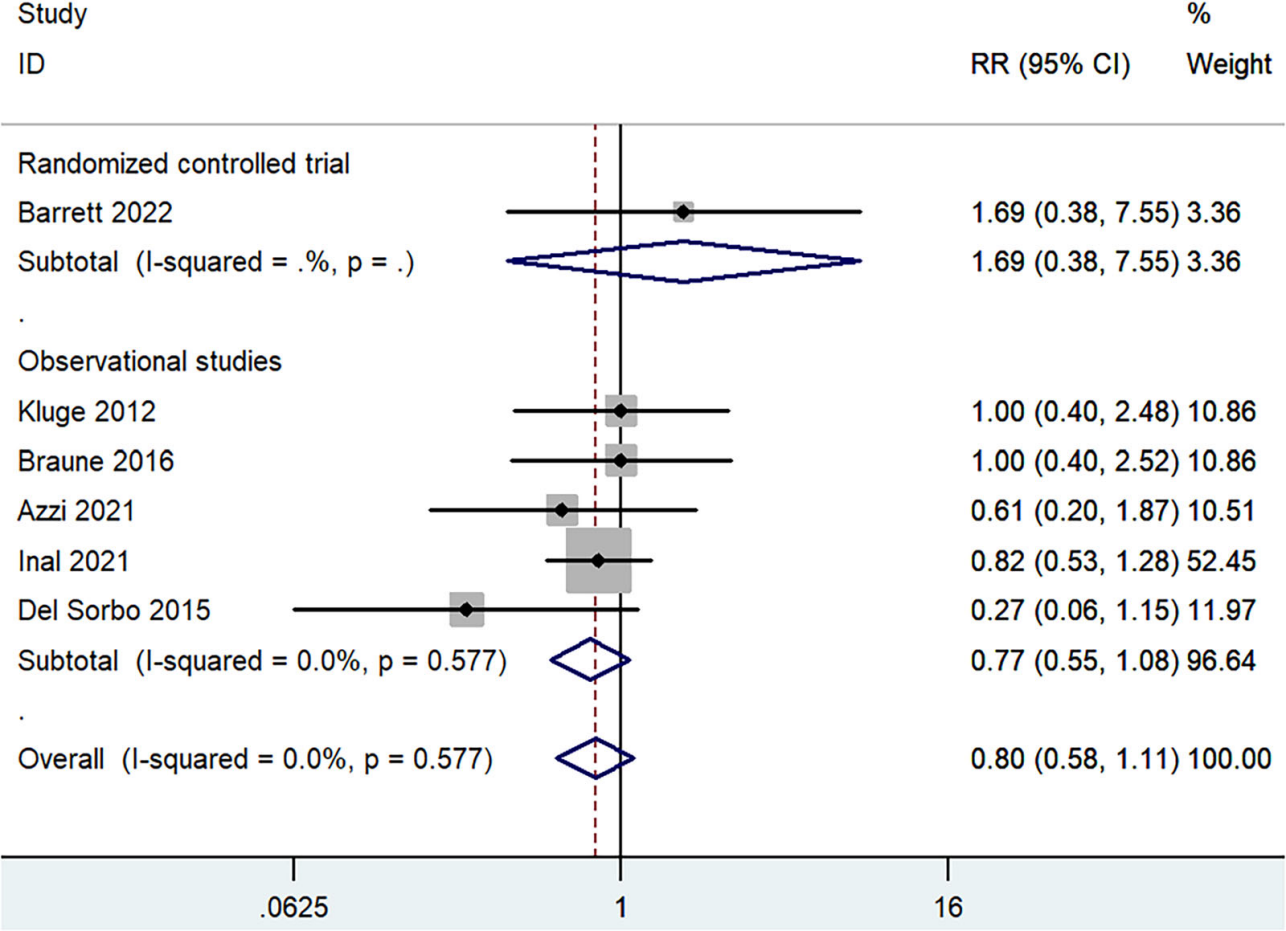
Forest plot for overall mortality within studies targeted patients with ARF secondary to COPD.

All the included studies reported data for mortality. Three RCTs [18–20] were done on ARDS or acute hypoxic respiratory failure patients who were mechanically ventilated, either low tidal volume or inverse ratio ventilation. Within the three RCTs, no statistically significant difference was observed in overall mortality between the ECCO_2_R group and control group (RR 1.05, 95% CI 0.83 to 1.32, *p* = 0.70, I^2^ =0.0%, Figure 2). The other RCT [21] and observational studies [22–26] targeted patients with ARF secondary to COPD. Pooled result of the five studies also showed similar overall mortality (RR 0.80, 95% CI 0.58 to 1.11, *p* = 0.19, I^2^ =0.0%, Figure 3), and the result was largely dependent on observational studies (RR 0.77, 95% CI 0.55 to 1.08, *p* = 0.13, I^2^ =0.0%, Figure 3). And in the subgroup analysis of mortality in patients with ARF secondary to COPD (Table 3), there was also no significant difference in 28-day mortality (RR 0.88, 95% CI 0.60 to 1.30, *p* = 0.53), 90-day mortality (RR 0.93, 95% CI 0.49 to 1.75, *p* = 0.82), ICU mortality (RR 1.02, 95% CI 0.07 to 15.73, *p* = 0.99) or hospital mortality (RR 0.99, 95% CI 0.25 to 4.01, *p* = 0.99). Similar mortality rates for ECCO_2_R and traditional therapy were also found in the VV-ECCO_2_R subgroup and patients older than 60 years old (RR 0.78, 95% CI 0.55 to 1.10, *p* = 0.16). Furthermore, no significant difference was observed between the two groups based on the severity of P/F ratio (Table 4).

**Table 3.** Subgroup analysis of mortality in patients with ARF secondary to COPD.

	No. of studies	RR (95% CI)	P	I^2^
Overall	6	0.80 (0.58, 1.11)	0.19	0.0%
28-day mortality	4	0.88 (0.60, 1.30)	0.53	0.0%
90-day mortality	3	0.93 (0.49, 1.75)	0.82	0.0%
≥6-month mortality	1	/	/	/
ICU mortality	2	1.02 (0.07, 15.73)	0.99	66.8%
Hospital mortality	3	0.99 (0.25, 4.01)	0.99	57.4%
Modality of AV-ECCO_2_R	1	/	/	/
Modality of VV-ECCO_2_R	5	0.78 (0.55, 1.10)	0.16	0.0%
Mean age (years) ≤60	1	/	/	/
Mean age (years) >60	5	0.78 (0.55, 1.10)	0.16	0.0%

ARF: acute respiratory failure; COPD: chronic obstructive pulmonary disease; ICU: intensive care unit; ECCO_2_R: extracorporeal carbon dioxide removal.

**Table 4.** Subgroup analysis of outcomes based on the severity of P/F ratio.

Group	Mortality				Length of ICU stay (days)				Length of hospital stay (days)			
	RCTs		Observational studies		RCTs		Observational studies		RCTs		Observational studies	
	Statistic (RR) (95%CI)	P	Statistic (RR) (95%CI)	P	Statistic (WMD) (95%CI)	P	Statistic (WMD) (95%CI)	P	Statistic (WMD) (95%CI)	P	Statistic (WMD) (95%CI)	P
**All**	1.05 (0.83, 1.32)	0.70	0.77 (0.55, 1.08)	0.13	1.57 (-0.73, 3.87)	0.18	−4.25 (-6.62, −1.87)	0.01	4.44 (0.62, 8.25)	0.02	−0.40 (-7.50, 6.71)	0.91
**Severity of P/F ratio**												
P/F ratio <150	1.04 (0.82, 1.32)	0.73	/	/	0.94 (-1.46, 3.34)	0.44	/	/	3.56 (-0.48, 7.60)	0.08	/	/
P/F ratio >150	/	/	0.74 (0.42, 1.32)	0.31			−4.16 (-7.13, −1.20)	0.01	/	/	1.79 (-5.70, 9.28)	0.64

Group	Intubation rate				Tracheotomy rate				Mechanical ventilation days (d)			
	RCTs		Observational studies		RCTs		Observational studies		RCTs		Observational studies	
	Statistic (RR) (95%CI)	P	Statistic (RR) (95%CI)	P	Statistic (RR) (95%CI)	P	Statistic (RR) (95%CI)	P	Statistic (SMD) (95%CI)	P	Statistic (SMD) (95%CI)	P
All	/	/	0.37 (0.24, 0.58)	0.01	/	/	0.48 (0.27, 0.85)	0.01	/	/	−0.43 (-0.66, -0.21)	0.01
**Severity of P/F ratio**												
P/F ratio <150	/	/	/	/	/	/	/	/	/	/	/	/
P/F ratio >150	/	/	0.42 (0.26, 0.70)	0.01	/	/	0.45 (0.14, 1.45)	0.18	/	/	−0.16 (-0.58, 0.26)	0.47

### 3.4. Length of ICU and hospital stay

All the included studies provided information on the length of ICU stay. Within RCTs targeted ARDS or acute hypoxic respiratory failure patients, there was a similar duration of ICU stay between ECCO_2_R group and control group (WMD 1.57, 95% CI −0.73 to 3.87, *p* = 0.18, I^2^ =37.0%) (Supplementary File 3: Figure S1a). However, within observational studies, treatment with ECCO_2_R significantly reduced length of ICU stay and the WMD was −4.25d with a 95% CI from −6.62 to −1.87 (*p* < 0.01), with no heterogeneity (I^2^ =0.0%) (Supplementary File 3: Figure S1b). And within observational studies, in ARF patients with P/F ratio beyond 150, we found that ECCO_2_R therapy was also significantly associated with a shorter length of ICU stay (WMD −4.16, 95% CI −7.13 to −1.20, *p* = 0.01) (Table 4).

A total of 4 RCTs and 4 observational studies described the length of hospital stay. The pooled results were shown in Supplementary File 3: Figure S2a and Figure S2b. Pooled results showed that the treatment of ECCO_2_R was associated with a longer length of hospital stay in patients with ARF secondary to ARDS or acute hypoxic respiratory failure (WMD 4.44, 95% CI 0.62 to 8.25, *p* = 0.02, I^2^ =10.5%). And the same result was observed in ARF patients secondary to COPD (WMD 3.91, 95% CI 0.96 to 6.85, *p* = 0.01, I^2^ =26.3%).

### 3.5. Intubation rate and tracheotomy rate

Data on the intubation rate were extracted and pooled from four observational studies, and target population of these studies are all patients with ARF secondary to COPD exacerbations. A total of 97 patients used ECCO_2_R during NIV and 77 patients had avoided intubation after the treatment of ECCO_2_R. And compared with control group, the pooled estimate suggested a lower intubation rate (RR 0.37, 95% CI 0.24 to 0.58, *p* < 0.01, I^2^ =33.7%) in ARF patients treated with the ECCO_2_R system (Supplementary File 3: Figure S3). Three observational trials reported data on the tracheotomy rate. Among 72 patients used ECCO_2_R during NIV, a total of 59 patients had avoided tracheostomy, and a lower tracheotomy rate was also observed in the ECCO_2_R group (RR 0.48, 95% CI 0.27 to 0.85, *p* = 0.01, I^2^ =19.7%) (Supplementary File 3: Figure S4).

### 3.6. Mechanical ventilation support and ventilator-free days

A total of dix studies (2 RCTs and four observational studies) including 592 patients reported data on mechanical ventilation days (d). Only one RCT targeted patients with ARF secondary to ARDS, which could not be estimated due to lack of enough data. However, the duration of mechanical ventilation was shorter in the ECCO_2_R group in comparison to the control group in ARF patients with AECOPD (SMD −0.43 d, 95% CI −0.66 to −0.21, *p* < 0.01, I^2^ = 19.3%) (Supplementary File 3: Figure S5). In addition, 2 RCTs provided information on ventilator-free days (VFDs). VFDs to 28 and 60 days were measured in the Xtravent study [18], and VFDs to 28 was measured in a REST RCT [19]. No significant difference was observed in VFDs to 28 or 60 days between the ECCO_2_R and control groups (WMD −0.74 d, 95% CI −3.41 to 1.92, *p* = 0.59, I^2^=37.1%) (Supplementary File 3: Figure S6).

### 3.7. Respiratory parameters

Respiratory parameters analysed in this meta-analysis included pH, PaCO2, PaO2, and respiratory rate. Only one RCT and one observational study reported data on PaO2/FiO2, which makes it difficult to analyse. With no difference in baseline characteristics between the control and intervention groups in these respiratory parameters, we collected data on the degrees of respiratory parameters after the treatment to reflect their improvement. As shown in the Supplementary File 3: Figure S7, compared to control group, treatment with ECCO_2_R could improve pH in ARF patients secondary to COPD (WMD 0.04, 95% CI 0.01 to 0.07, *p* = 0.01, I^2^ =72.6%), but not in ARDS or acute hypoxic respiratory failure patients (WMD 0.01, 95% CI −0.02 to 0.04, *p* = 0.38, I^2^ =67.6%). And the mean pH after treatment with ECCO_2_R in patients with ARF secondary to COPD fluctuated from 7.34 to 7.41. An improvement of PaO2 (WMD 0.75, 95% CI 0.19 to 1.32, *p* = 0.01, I^2^ =9.9%), respiratory rate (WMD −4.51, 95% CI −6.60 to −2.42, *p* < 0.01, I^2^ =0.0%) and PaCO2 (WMD −10.31, 95% CI −19.93 to −0.70, *p* = 0.04, I^2^ =85.7%) was also observed in patients with ARF secondary to COPD by ECCO_2_R therapy.

### 3.8. Adverse events

With the exception of the study conducted by İnal et al. [26], all included studies reported on ECCO_2_R-related complications, such as clotting within the circuit, pump malfunction, bleeding, transient ischemia of lower limb, and even lower limb amputation. Six studies reported ECCO_2_R-related complication rates in excess of 20% [19,20,22–25]. We analysed the incidence of major complications, including major bleeding, pneumothorax and so on, and we found a higher major complication rate of the ECCO_2_R therapy (RR 3.35, 95% CI 2.08 to 5.42, *p* < 0.01, I^2^ =17.1%), after the analysis of two RCTs (Supplementary File 3: Figure S8). The details of the incidence of adverse events were shown in Table 2.

### 3.9. Cost

Two studies reported costs in different ways, including total costs in 2 studies [20,25], and daily cost in 1 study [20]. Kluge et al. [25] indicated a lower cost with ECCO_2_R therapy compared to invasive mechanical ventilation. However, the result was opposite in study conducted by Morris et al. [20]. Meta-analysis could not be estimated due to lack of enough studies.

### 3.10. Publication bias

Begg’s funnel plots and Egger’s test (with respect to mortality, length of ICU or hospital stay, respectively) were used to analyse publication bias. *p* > 0.05 means that there is no obvious publication bias. We give priority to the results of Egger’s test when the results of Begg’s and Egger’s tests are inconsistent. All studies were arranged roughly symmetrically around the centerline, and *p* > 0.05 could be observed in all the results of Egger’s test, which indicates that no evidence of obvious publication bias was detected (Supplementary File 4).

## 4. Discussion

### 4.1. Key findings

We performed a systematic review and meta-analysis of the literature and identified 9 original articles (4 RCTs and five observational studies) to evaluate the efficacy of ECCO_2_R among more than 1100 patients with ARF. We found that patients receiving ECCO_2_R therapy had a similar risk of mortality compared with those in the control group, neither in RCTs targeted ARDS or acute hypoxic respiratory failure patients nor in observational studies targeted patients with ARF secondary to COPD. ECCO_2_R therapy was associated with a longer length of hospital stay both in ARF patients secondary to ARDS or acute hypoxic respiratory failure and COPD. And a reduction in the length of ICU stay was only obtained in observational studies. Instead, ECCO_2_R was associated with a lower intubation rate and tracheotomy rate, and shorter mechanical ventilation days in ARF patients with COPD. In addition, an improvement in pH, PaO2, respiratory rate and PaCO2 was observed in ARF patients with COPD exacerbations by ECCO_2_R therapy. However, the ECCO_2_R-related complication rate was high (more than 20%) in six of the included studies.

### 4.2. Comparison with previous studies

Several systematic reviews have reviewed the potential risk and benefits of ECCO_2_R in patients with ARF. A systematic review including 2 RCTs and 12 observational studies assessed mortality, length of stay, VFDs, organ failure-free days, and complication rates of ECCO_2_R in patients with ARF secondary to ARDS [27]. Another systematic review conducted by Sklar 2015 reviewed the risks, efficacy, and benefits of ECCO_2_R in ARF patients with COPD [28]. However, there are still no meta-analyses evaluating the effectiveness of ECCO_2_R in ARF patients. Our present meta-analysis included data from 4 RCTs and 5 observational studies, comprehensively comparing the efficacy and benefits of ECCO_2_R therapy in patients with ARF for the first time. Such a comprehensive meta-analysis would be more likely to accurately represent the effects of ECCO_2_R therapy in patients with ARF.

### 4.3. Clinical implications and future studies

ECCO_2_R, as an experimental adjunct to mechanical ventilation, has been utilized to avoid intubation or tracheotomy, and reduce the length of invasive ventilation. It also has been proven to have advantages in removing carbon dioxide and correcting respiratory acidosis in patients with ARF [29]. Currently, an increasing number of studies support the role of ECCO_2_R in the treatment of life-threatening critically ill critical illnesses, especially ARF [10,30]. However, there are only a few high-quality studies comparing ECCO_2_R with other therapies for ARF. Most studies elucidating the efficacy of ECCO_2_R are case reports or case series with small number [31–33]. Thus, further high-quality prospective controlled studies are urgently needed to compare ECCO_2_R with other therapies for patients with ARF. And several ongoing randomized clinical trials on ECCO_2_R treatment for ARF are list in Table 5.

**Table 5.** Ongoing randomized clinical trials on ECCO_2_R therapy for patients with ARF.

Status	NCT	Study	Target population
Active, recruiting	NCT04842344	A prospective randomized controlled trial of extracorporeal carbon dioxide removal (ECCO_2_R) in the treatment of acute exacerbation of chronic obstructive pulmonary disease with severe hypercapnia	COPD
Active, recruiting	NCT03255057	A prospective, multi-center, randomized, controlled, pivotal trial to validate the safety and efficacy of the Hemolung® respiratory assist system for COPD patients experiencing an acute exacerbation requiring ventilatory support	COPD
Active, recruiting	NCT03525691	Enhanced lung protective ventilation with extracorporeal CO_2_ removal during acute respiratory distress syndrome	ARDS
Not yet recruiting	NCT04582799	Extracorporeal carbon dioxide removal for acute decompensation of chronic obstructive pulmonary disease: A randomized clinical trial (The ORION Study)	COPD
Not yet recruiting	NCT04147104	ECCO_2_R to facilitate early liberation from mechanical ventilation inpatients with COPD acute exacerbation	COPD
Not yet recruiting	NCT03584295	A multicenter randomized-controlled trial of extracorporeal CO_2_ removal to facilitate early extubation compared to invasive mechanical ventilation in patients with severe acute exacerbation of COPD (X-COPD)	COPD
Not yet recruiting	NCT04903262	Strategy of ultra-protective lung ventilation with extracorporeal CO_2_ removal for new-onset moderate ARDS: A prospective multicenter randomized clinical trial	ARDS

ECCO_2_R: extracorporeal carbon dioxide removal; COPD: chronic obstructive pulmonary disease; ARDS: acute respiratory distress syndrome.

Currently, combined ECCO_2_R and continuous renal replacement therapy (CRRT) (ECCO_2_R-CRRT) in patients with ARF and acute kidney injury (AKI) is an interesting approach that was recently developed [34,35]. ARDS, exacerbation of COPD and mechanical ventilation are independently associated with an increased risk of AKI [36–38]. Studies have noted that ECCO_2_R-CRRT could provide advantages for both the regulation of CO_2_ extraction and potential renal metabolic compensation [35,39–41]. However, there are still no multiple prospective controlled studies with large number to confirm the safety and efficacy of ECCO_2_R-CRRT, and further well-designed studies are needed in the future.

### 4.4. Strengths and limitations

This meta-analysis comprehensively evaluated the effect of ECCO_2_R in patients with ARF. Our search strategy was broad and included all the relevant studies with no publication year restrictions. It included data from more than 1100 patients and multiple countries including France, Turkey, Germany, Italy, Austria and the United Kingdom. A variety of outcomes such as mortality, length of hospital and ICU stay, intubation and tracheotomy rate, mechanical ventilation days, VFDs, respiratory parameters, reported adverse events, and cost were analysed in our meta-analysis. In addition, no obvious publication bias of our results was observed through Begg’s funnel plots and Egger’s test. Furthermore, two independent investigators thoroughly evaluated methodological quality.

However, several limitations also existed in this meta‑analysis. First, although a total of 9 studies were included in this meta-analysis, only four eligible studies were RCTs and six of the included studies were small (less than 60 patients). Second, outcomes are largely dependent on observational studies, which might contribute to allocation or selection bias. In addition, insufficient data were available to evaluate VFDs, adverse events and cost. Third, moderate or significant heterogeneity existed in outcomes such as length of ICU stay, and respiratory parameters. A random effects model was used for potential heterogeneity, and subgroup analysis was performed when data were available; however, only weak hypothesis-generating evidence could be provided. Fourth, only published studies with selective databases, but not unreported outcomes were included for data analysis, which could possibly result in reporting bias. Regardless of these limitations, we minimized bias throughout the analysis by strict method identification, data selection, statistical analysis, and subgroup analysis. These steps should strengthen the stability and accuracy of our meta-analysis.

## 5. Conclusions

Our findings from both RCTs and observational studies did not confirm a significant beneficial effect of ECCO_2_R therapy on mortality. The reducing of length of ICU stay was only obtained in observational studies. Instead, lower intubation rate and tracheotomy rate, and shorter mechanical ventilation days were observed in the ECCO_2_R group in ARF patients with COPD. However, Improvements of ECCO_2_R technology to decrease ECCO_2_R-related complications are necessary, and further larger high-quality RCTs are desirable to strengthen the efficacy of ECCO_2_R for patients with ARF.

## Supplementary Material

Supplemental Material

Supplemental Material

Supplemental Material

Supplemental Material

Supplemental Material

## Data Availability

All data generated or analyzed during this study are included in this article and its supplementary information files.
