# Supplementary material for: Extracorporeal carbon dioxide removal for patients with acute respiratory failure: a systematic review and meta-analysis
Source: Ann Med. 2023 Mar 1;55(1):746–59. doi: 10.1080/07853890.2023.2172606 (PMC9980035; doi:10.1080/07853890.2023.2172606)
Supplement: Supplemental Material [file IANN_A_2172606_SM0970.docx]

Supplementary Table 1. Inclusion criteria and exclusion criteria of participants in the included studies.

| Study | Inclusion criteria | Exclusion criteria |
| --- | --- | --- |
| Bein et al | 1) presence of ARDS according to the American-European Consensus Conference defined by bilateral infiltrates on chest X-ray, and a PaO2/FIO2 <200 present for at least 2 h. At the time of screening patients could not have any evidence of left ventricular failure; 2) age ≥18 years; 3) history of mechanical ventilation <7 days); 4) plateau pressure >25 cmH_2_O at defined ventilator settings (PEEP/FIO2-table + V T = 6 ml/kg); 5) absence of severe hemodynamic instability with high demand for vasopressors (mean arterial pressure ≥70 mmHg with continuous norepinephrine infusion ≤0.4 μg/kg/min) | Decompensated heart insufficiency, acute coronary syndrome, severe chronic obstructive pulmonary disease, advanced malignancy with life expectancy <6 months, chronic dialysis treatment, lung transplant patients, proven heparin-induced thrombocytopenia (HIT), morbid obesity (body mass index >40 kg/m^2^), cirrhosis of the liver Child Class ≥B (Child–Pugh scores ≥7), or acute fulminant hepatic failure, severe peripheral arterial occlusive disease, absence of limb doppler pulse, and acute brain injury (Glasgow Coma Scale ≤9) |
| McNamee et al | 1)age ≥18 years; 2) acute and potentially reversible cause of moderate to severe hypoxemic respiratory; 3) receiving invasive mechanical ventilation using at least 5 cm H2O of positive end-expiratory pressure (PEEP); 4) within 48 hours of onset of hypoxemia, defined as a ratio of the partial pressure of oxygen in arterial blood to the fractional inspired concentration of oxygen (PaO2/FiO2) of less than 150 mmHg | Receiving invasive mechanical ventilation for more than 7 days, contraindication to limited systemic anticoagulation with heparin, untreated pulmonary embolism, pleural effusion or pneumothorax, or acute respiratory failure fully explained by left ventricular failure or fluid overload |
| Morris et al | All patients were screened for ARDS. ARDS was defined by the presence of all the following: P(a/A) O_2_<0.2, bilateral chest radiographic infiltrates, CTH <50 ml/cm H_2_0, and Pw ≤15 mmHg (or no clinical evidence of heart failure) | Patients <12 and >65 years of age and those who had already undergone mechanical ventilation (ARDS duration) for >21 days |
| Barrett et al | Age ≥18 years; had a history of COPD presenting with AECOPD and with a persisting pH < 7.30 due to hypercapnia after initial medical therapy and at least 1 h of NIV | Acute multiple organ failure, intolerance, allergy or contraindication to heparin or a contraindication to NIV |
| Azzi et al | No improvement or worsening of respiratory acidosis after NIV treatment, and no improvement of respiratory distress signs or decreased level of consciousness | Severe hypoxemia (FIO2 >40% for an oxygen saturation ≥90% with NIV), contraindications to anticoagulant therapy, contraindications to continuation of active treatment for reasons of futility |
| Braune et al | Consecutive patients older than 18 years with hypercapnic ventilatory failure requiring NIV | Severe hypoxemia (PaO2/FiO2 ratio <100 mmHg), pre-existing home NIV treatment, contraindications to anticoagulation, contraindication to continuation of active treatment for reasons of futility and failure to obtain consent |
| Del Sorbo et al | Patients older than 18 and younger than 90 years treated with NIV for acute hypercapnic respiratory failure due to exacerbation of COPD | Mean arterial pressure less than 60 mm Hg despite infusion of fluids and vasoactive drugs; contraindications to anticoagulation (i.e., any of the following: platelet count <30,000/mm^3^; prothrombin time-international normalized ratio >1.5); stroke or severe head trauma or intracranial arteriovenous malformation, or cerebral aneurysm, or CNS mass lesion within the previous 3 months; epidural catheter in place or expected to be positioned during the study; history of congenital bleeding diatheses; gastrointestinal bleeding within the 6 weeks prior to study entry; esophageal varices, chronic jaundice, cirrhosis, or chronic ascites; trauma; body weight greater than 120 kg; contraindication to continuation of active treatment; and failure to obtain consent |
| Kluge et al | All non-intubated patients who were treated with PECLA for acute hypercapnic respiratory failure | / |
| İnal et al | 1) persisting severe hypercapnic respiratory acidosis (pH < 7.15) despite optimized attempts of iMV for more than 3 h; 2) lung protective ventilation was required but hypercapnia was undesirable or contraindicated; 3) no contraindications for canulation and systemic anticoagulation; 4) hemodynamic status was manageable; 5) the underlying disease was reversable or no markers of poor short-term prognosis | Patients detected with both of COPD and ARDS diagnoses |
