# Supplementary material for: Extracorporeal carbon dioxide removal for patients with acute respiratory failure: a systematic review and meta-analysis
Source: Ann Med. 2023 Mar 1;55(1):746–59. doi: 10.1080/07853890.2023.2172606 (PMC9980035; doi:10.1080/07853890.2023.2172606)
Supplement: Supplemental Material [file IANN_A_2172606_SM0640.docx]

Search strategy terms

| PubMed, searched 30/4/2022 | |
| --- | --- |
| 1 | Extracorporeal carbon dioxide removal [All Fields] OR ECCO2R [All Fields] OR Extracorporeal lung assist [All Fields] OR Interventional lung assist [All Fields] |
| 2 | Acute respiratory failure [All Fields] OR Acute respiratory distress syndrome [All Fields] OR Chronic obstructive pulmonary disease [All Fields] |
| 3 | Combine #1 AND #2 |

| Embase, searched 30/4/2022 | |
| --- | --- |
| 1 | Extracorporeal carbon dioxide removal [ti/ab] OR ECCO2R [ti/ab] OR Extracorporeal lung assist [ti/ab] OR Interventional lung assist [ti/ab] |
| 2 | Acute respiratory failure [ti/ab] OR Acute respiratory distress syndrome [ti/ab] OR Chronic obstructive pulmonary disease [ti/ab] |
| 3 | Combine #1 AND #2 |

| Web of Science, searched 30/4/2022 | |
| --- | --- |
| 1 | TS = (Extracorporeal carbon dioxide removal) OR TS = (ECCO2R) OR TS = (Extracorporeal lung assist) OR TS = (Interventional lung assist) |
| 2 | TS = (Acute respiratory failure) OR TS = (Acute respiratory distress syndrome) OR TS = (Chronic obstructive pulmonary disease) |
| 3 | Combine #1 AND #2 |

| the Cochrane Library, searched 30/4/2022 | |
| --- | --- |
| 1 | Extracorporeal carbon dioxide removal [ti/ab/kw] OR ECCO2R [ti/ab/kw] OR Extracorporeal lung assist [ti/ab/kw] OR Interventional lung assist [ti/ab/kw] |
| 2 | Acute respiratory failure [ti/ab/kw] OR Acute respiratory distress syndrome [ ti or ab or kw] OR Chronic obstructive pulmonary disease [ ti or ab or kw] |
| 3 | Combine #1 AND #2 |
