# Supplementary material for: Extracorporeal carbon dioxide removal for patients with acute respiratory failure: a systematic review and meta-analysis
Source: Ann Med. 2023 Mar 1;55(1):746–59. doi: 10.1080/07853890.2023.2172606 (PMC9980035; doi:10.1080/07853890.2023.2172606)
Supplement: Supplemental Material [file IANN_A_2172606_SM9121.docx]

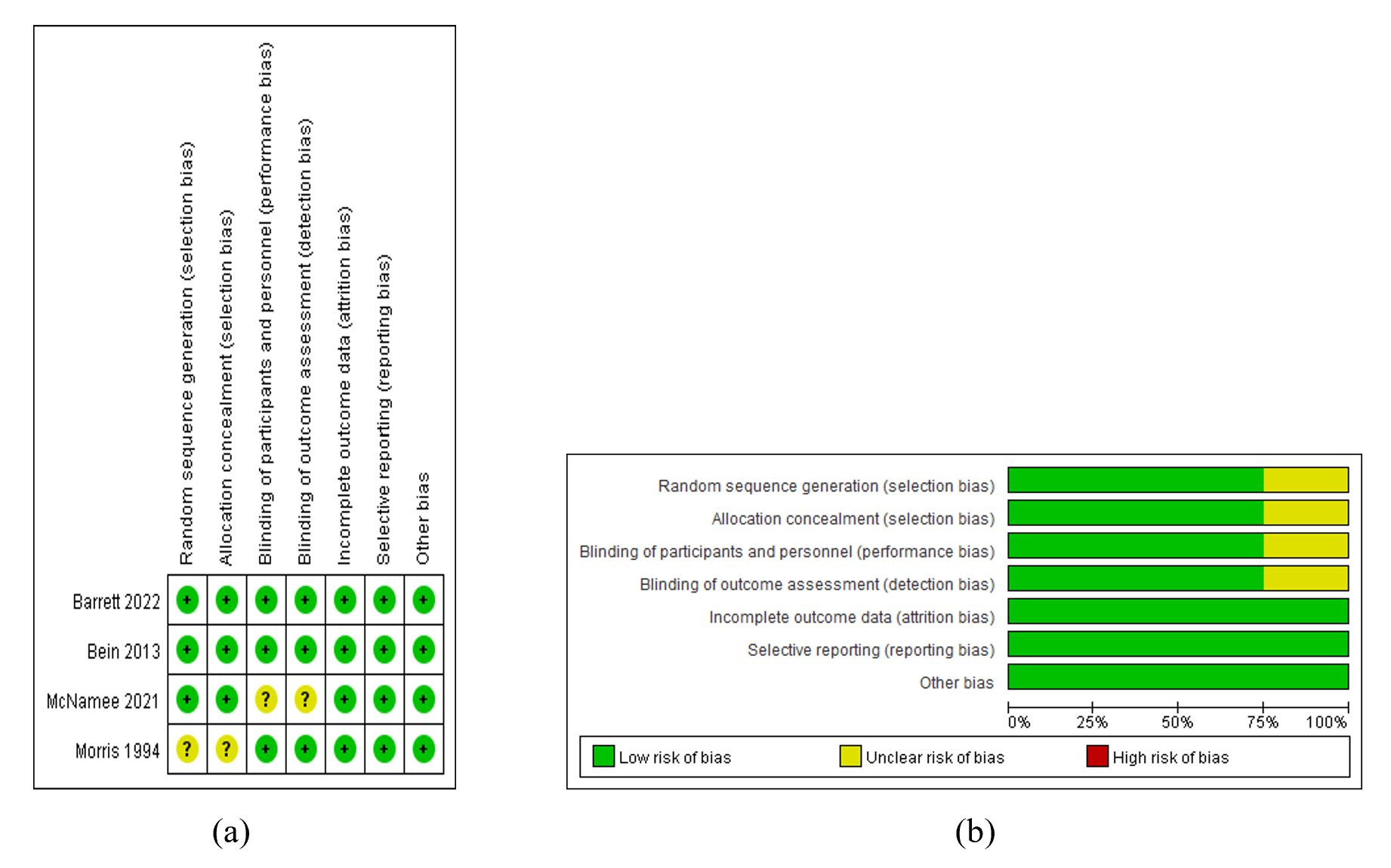


Figure 1. Results of quality assessment of the included RCTs. (a) Potential risk of bias of each included study. (b) Summarized risk of the included studies.

Table 1. Results of quality assessment of the included observational studies

| COHORT STUDIES | | | | | | | | | |
| --- | --- | --- | --- | --- | --- | --- | --- | --- | --- |
|  | Selection | | | | Comparability | | Outcome | | |
| Study | Representativeness of the exposed cohort | Selection of the non-exposed cohort | Ascertainment of exposure | Demonstration that outcome of interest was not present at start of study | Comparability main | Comparability additional factors | Assessment of outcome | Was follow-up long enough for outcomes to occur | Adequacy of follow up of cohorts |
| Azzi et al | **🟑** | **🟑** | **🟑** |  | **🟑** | ? |  | **🟑** | **🟑** |
| Del Sorbo et al | **🟑** | **🟑** | **🟑** |  | **🟑** | **🟑** | **🟑** | **🟑** | **🟑** |

| CASE-CONTROL STUDIES | | | | | | | | | |
| --- | --- | --- | --- | --- | --- | --- | --- | --- | --- |
|  | Selection | | | | Comparability | | Outcome | | |
| Study | Was the Case Definition Adequate | Representativeness of the Cases | Selection of Controls | Definition of Controls | Comparability main | Comparability additional factors | Ascertainment of Exposure | Was the same method used to determine exposure factors | Non-Response Rate |
| Braune et al | **🟑** | **🟑** | **🟑** | **🟑** | **🟑** | **🟑** | **🟑** | **🟑** | **🟑** |
| Kluge et al | **🟑** | **🟑** | **🟑** | **🟑** | **🟑** | **🟑** | **🟑** | **🟑** | **🟑** |
| İnal et al | **🟑** | **🟑** | **🟑** | **🟑** | **🟑** |  | **🟑** | **🟑** | **🟑** |
