# Supplementary material for: Extracorporeal carbon dioxide removal for patients with acute respiratory failure: a systematic review and meta-analysis
Source: Ann Med. 2023 Mar 1;55(1):746–59. doi: 10.1080/07853890.2023.2172606 (PMC9980035; doi:10.1080/07853890.2023.2172606)
Supplement: Supplemental Material [file IANN_A_2172606_SM9112.docx]

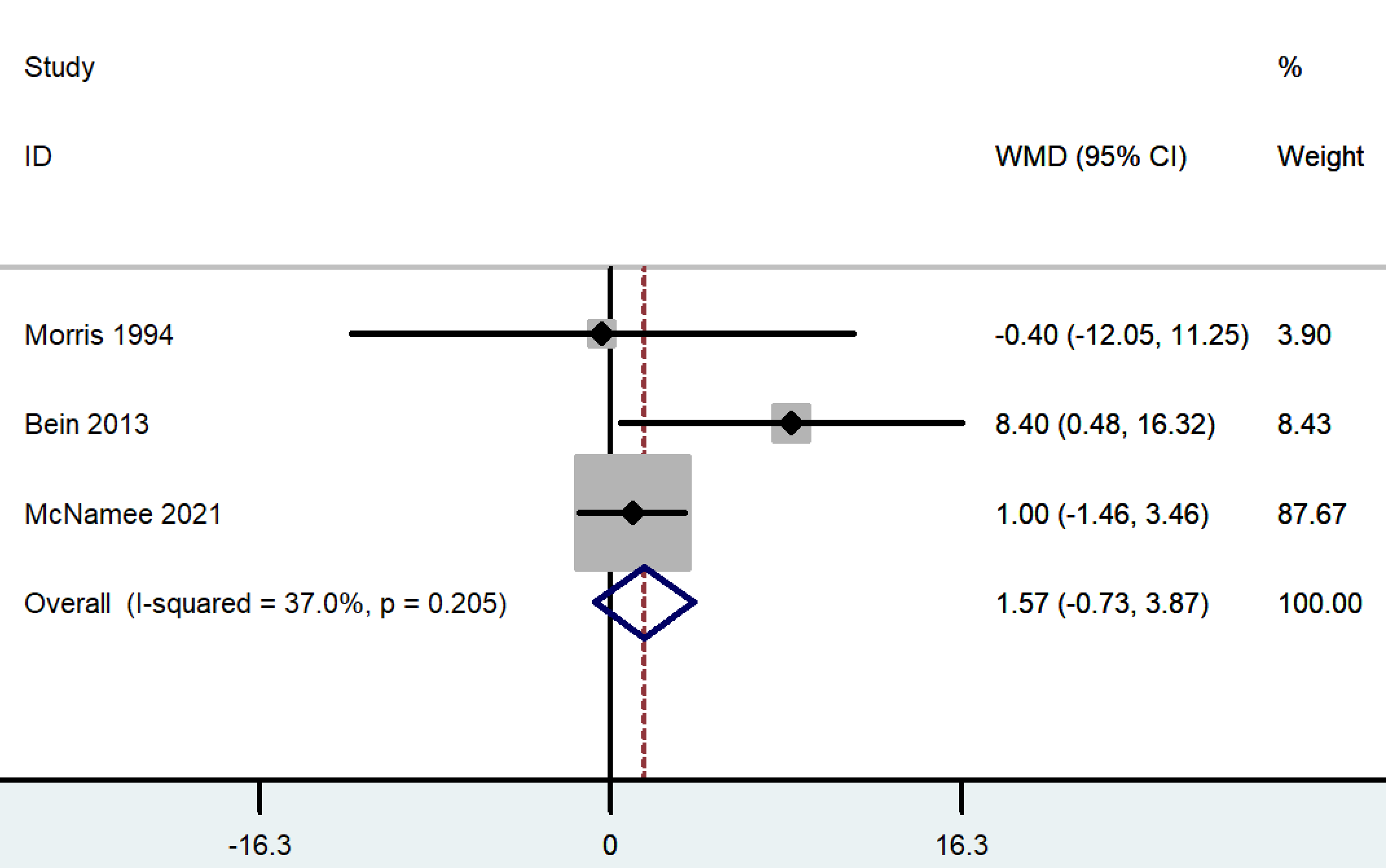


**Figure S1a.** Forest plot for length of ICU stay within RCTs targeted patients with ARF secondary to ARDS or acute hypoxic respiratory failure.


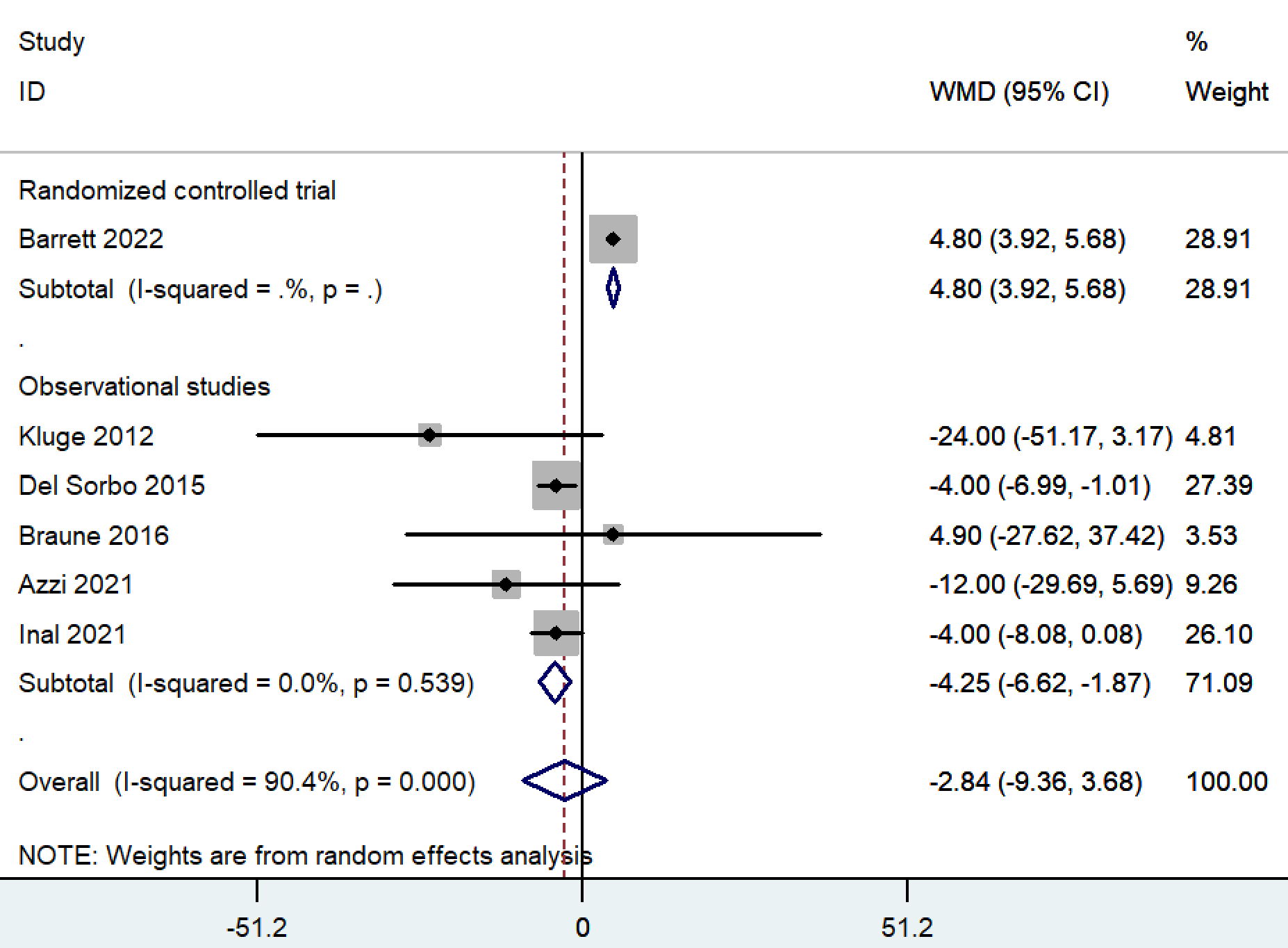


**Figure S1b.** Forest plot for length of ICU stay within studies targeted patients with ARF secondary to COPD.


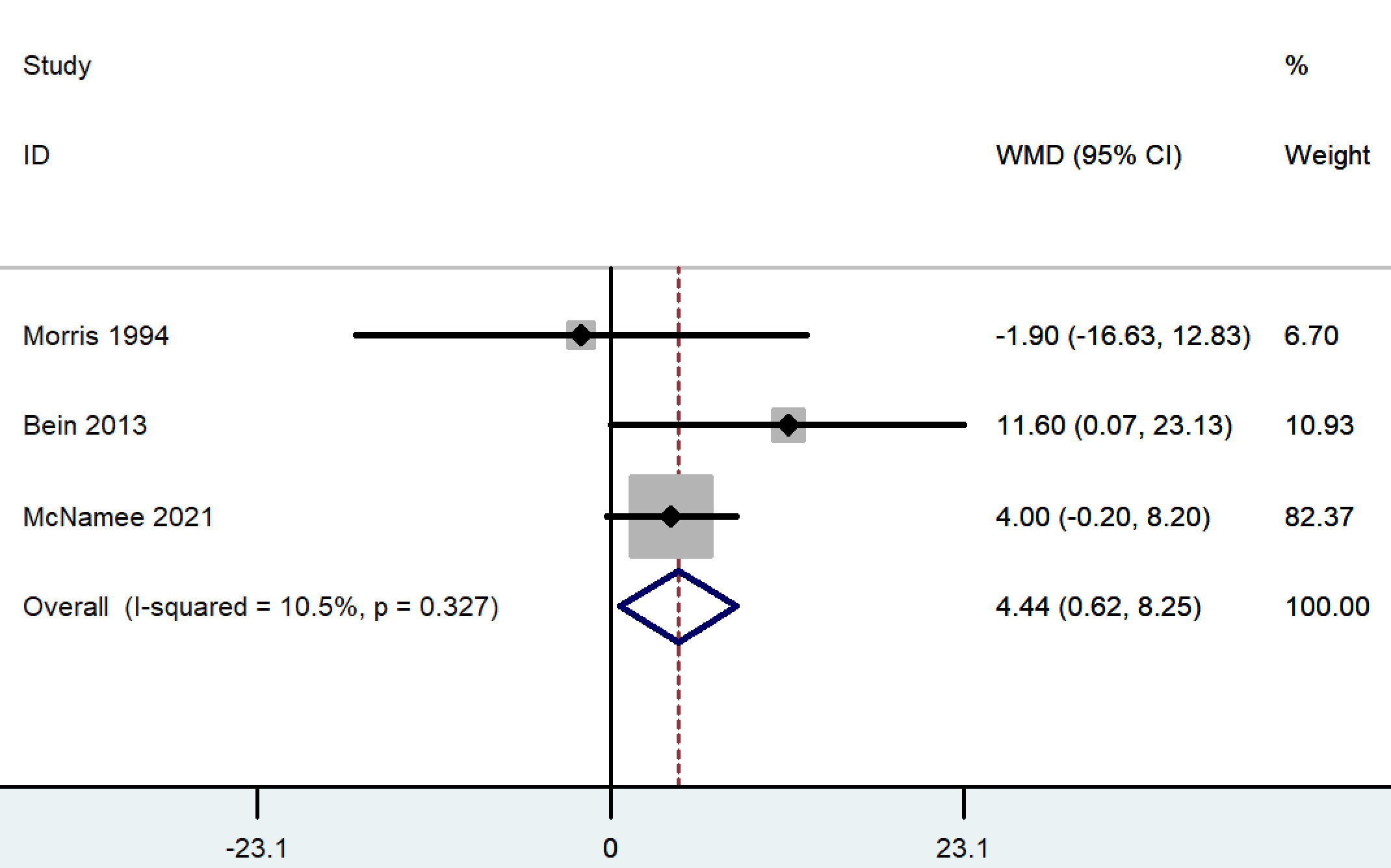


**Figure S2a.** Forest plot for length of hospital stay within RCTs targeted patients with ARF secondary to ARDS or acute hypoxic respiratory failure.


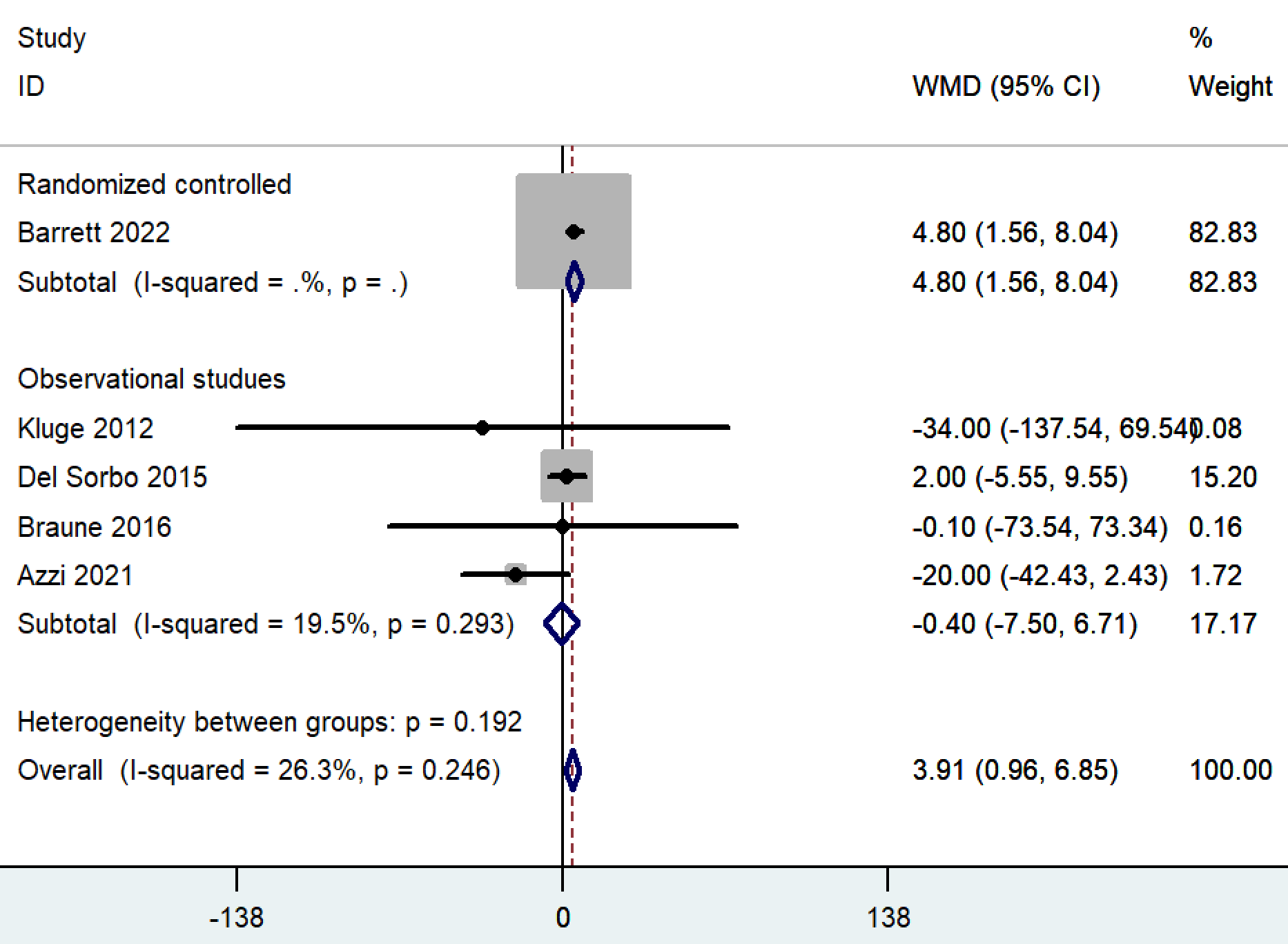


**Figure S2b.** Forest plot for length of hospital stay within studies targeted patients with ARF secondary to COPD.


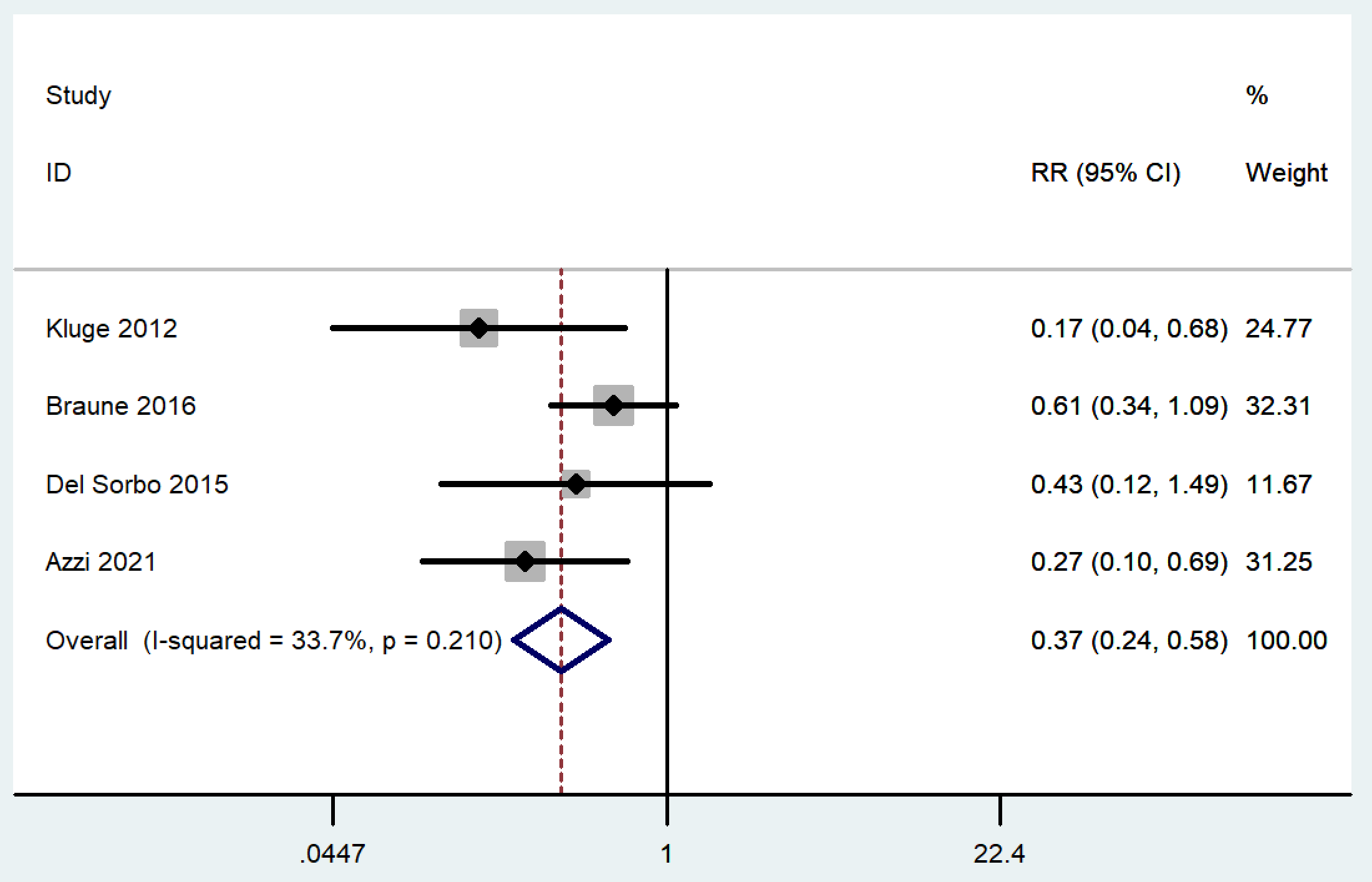


**Figure S3.** Intubation rate. (RR 0.37, 95% CI 0.24 to 0.58, P<0.01, I^2^ =33.7%)


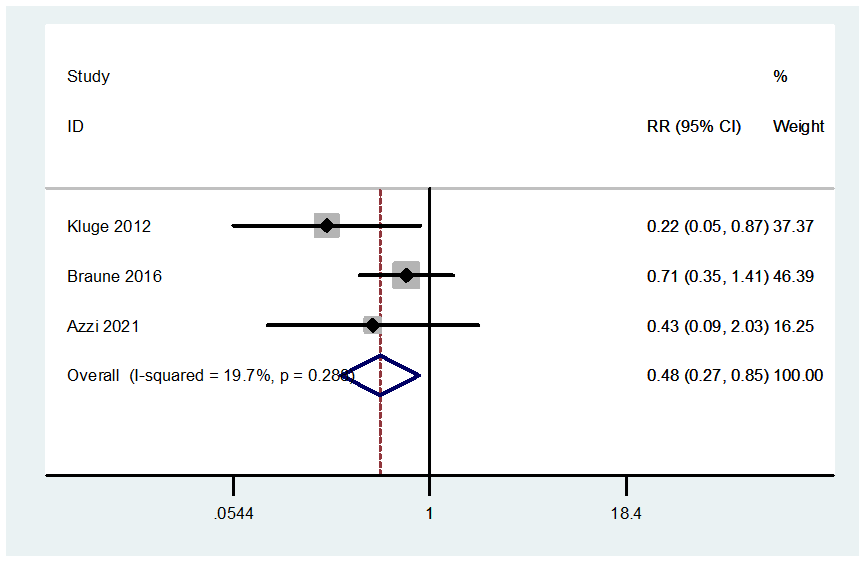


**Figure S4.** Tracheotomy rate. (RR 0.48, 95% CI 0.27 to 0.85, P=0.01, I^2^ =19.7%)


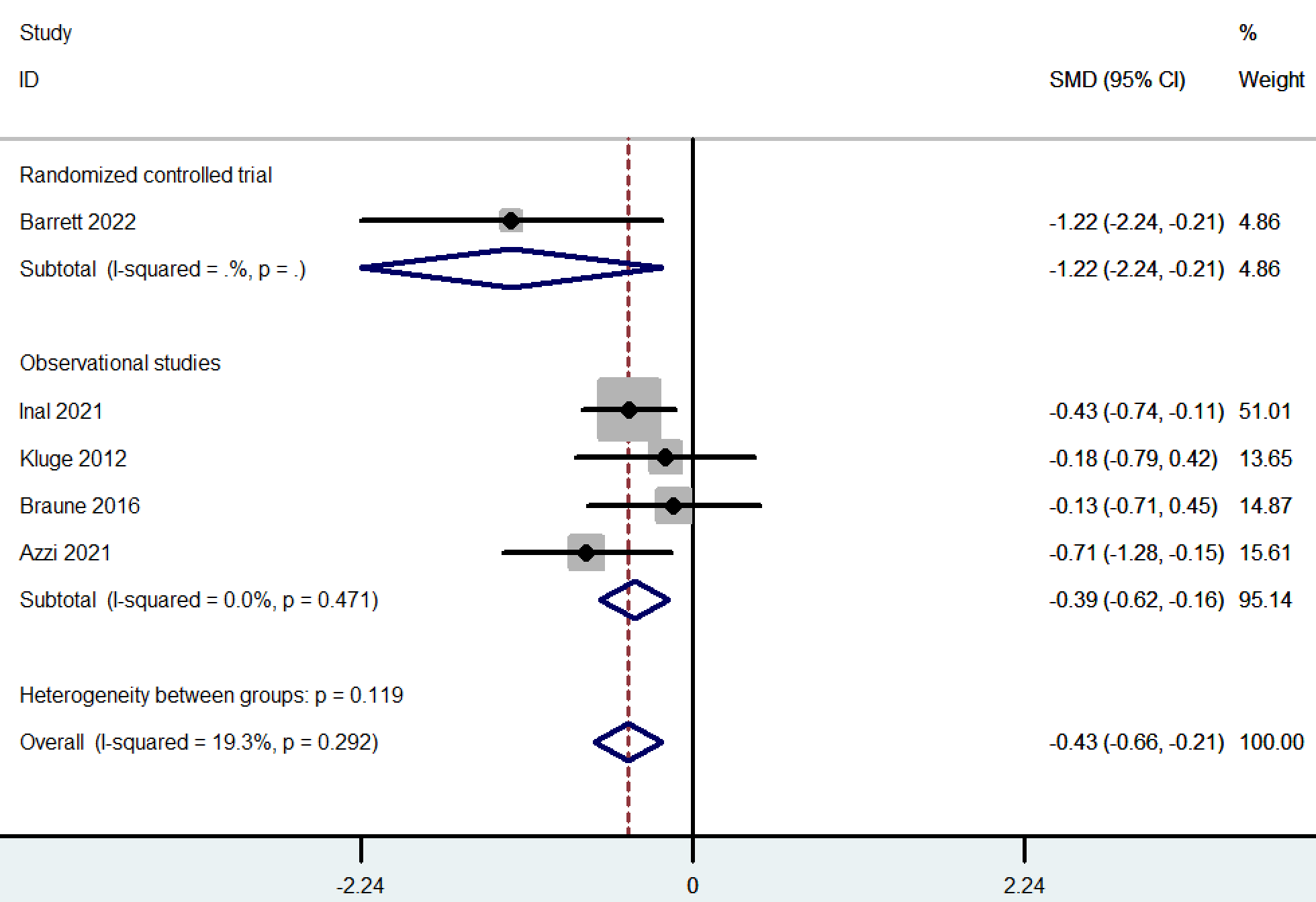


**Figure S5.** Mechanical ventilation days (d). (Overall: SMD -0.43 d, 95% CI -0.66 to -0.21, P<0.01, I^2^ = 19.3%; observational studies: SMD -0.39 d, 95% CI -0.62 to -0.16, P<0.01, I^2^ = 0.0%)


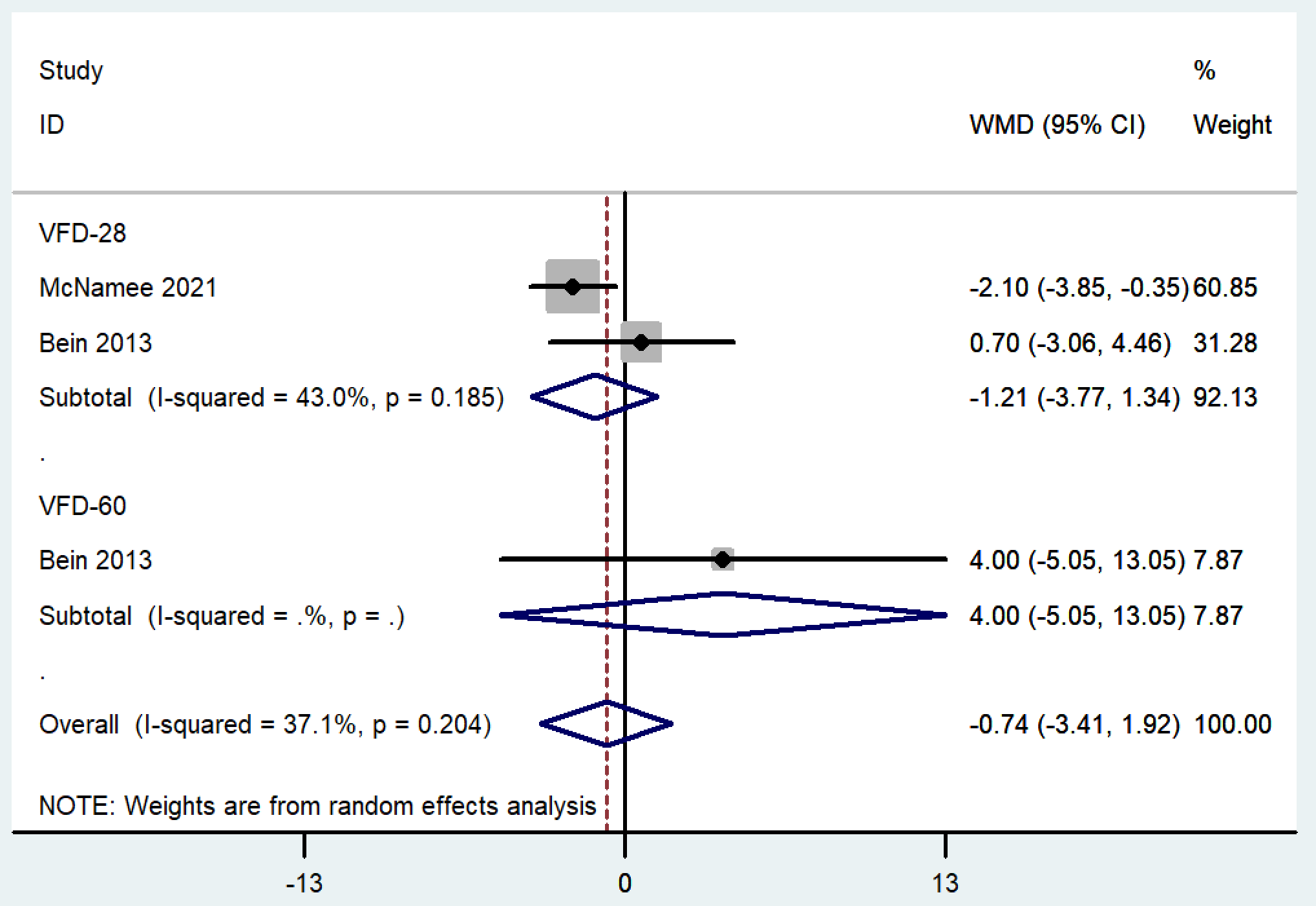


**Figure S6.** Ventilator-free days (d). (WMD -0.74 d, 95% CI -3.41 to 1.92, P=0.59, I^2^=37.1%) VFD-28: WMD -1.21 d, 95% CI -3.77 to 1.34, P=0.35, I^2^=43.0%


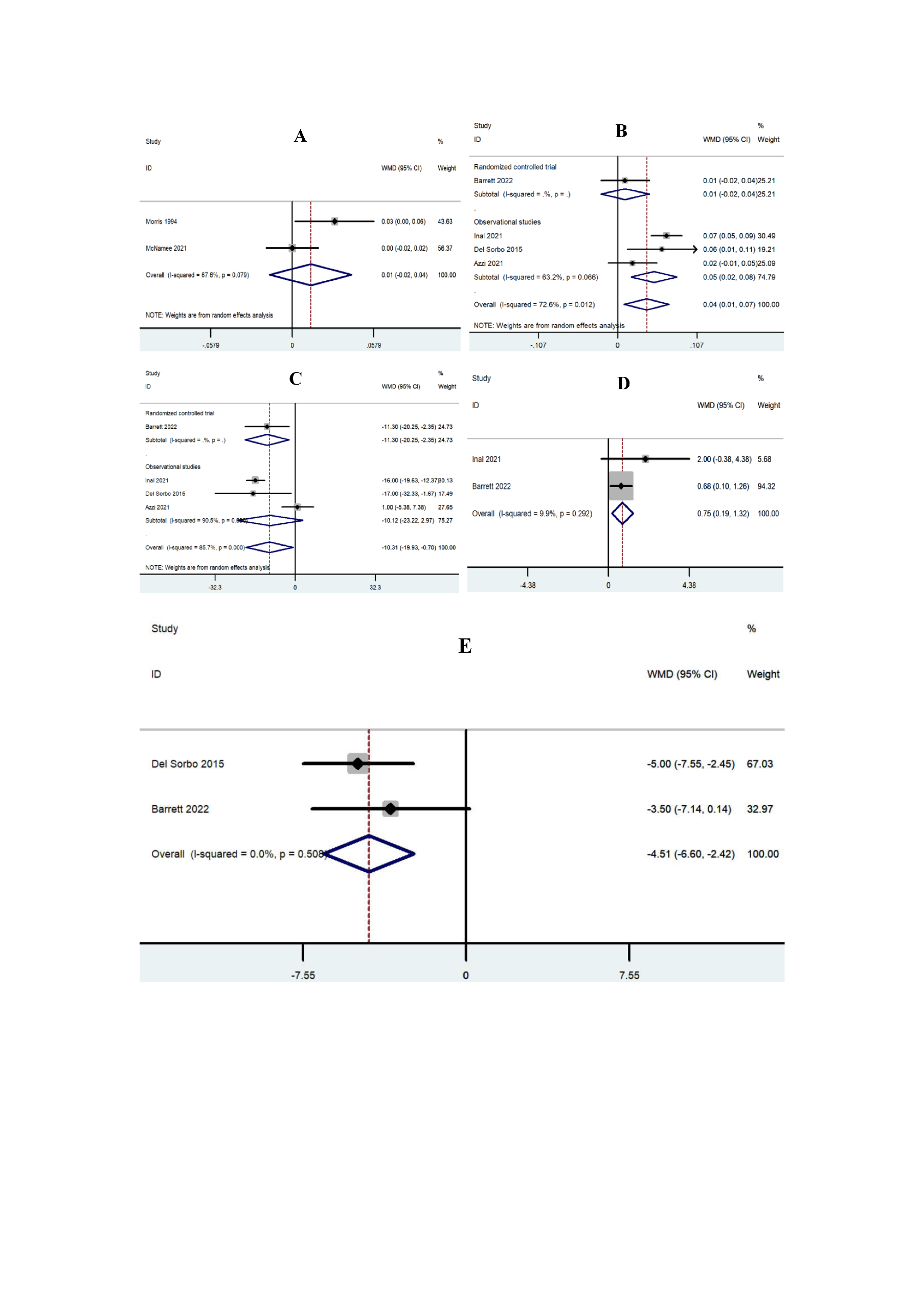


**Figure S7.** Respiratory parameters.

1. pH (2 RCTs targeted ARDS or acute hypoxic respiratory failure patients) (WMD 0.01, 95% CI -0.02 to 0.04, P=0.38, I^2^ =67.6%).
2. pH (studies targeted patients with ARF secondary to COPD) (Overall: WMD 0.04, 95% CI 0.01 to 0.07, P=0.01, I^2^ =72.6%; observational studies: WMD 0.05, 95% CI 0.02 to 0.08, P<0.01, I^2^ =63.2%).

(C) PaCO2 (WMD -10.31, 95% CI -19.93 to -0.70, P=0.04, I^2^ =85.7%).

(D) PaO2 (WMD 0.75, 95% CI 0.19 to 1.32, P=0.01, I^2^ =9.9%).

(E) Respiratory rate. (WMD -4.51, 95% CI -6.60 to -2.42, P<0.01, I^2^ =0.0%).


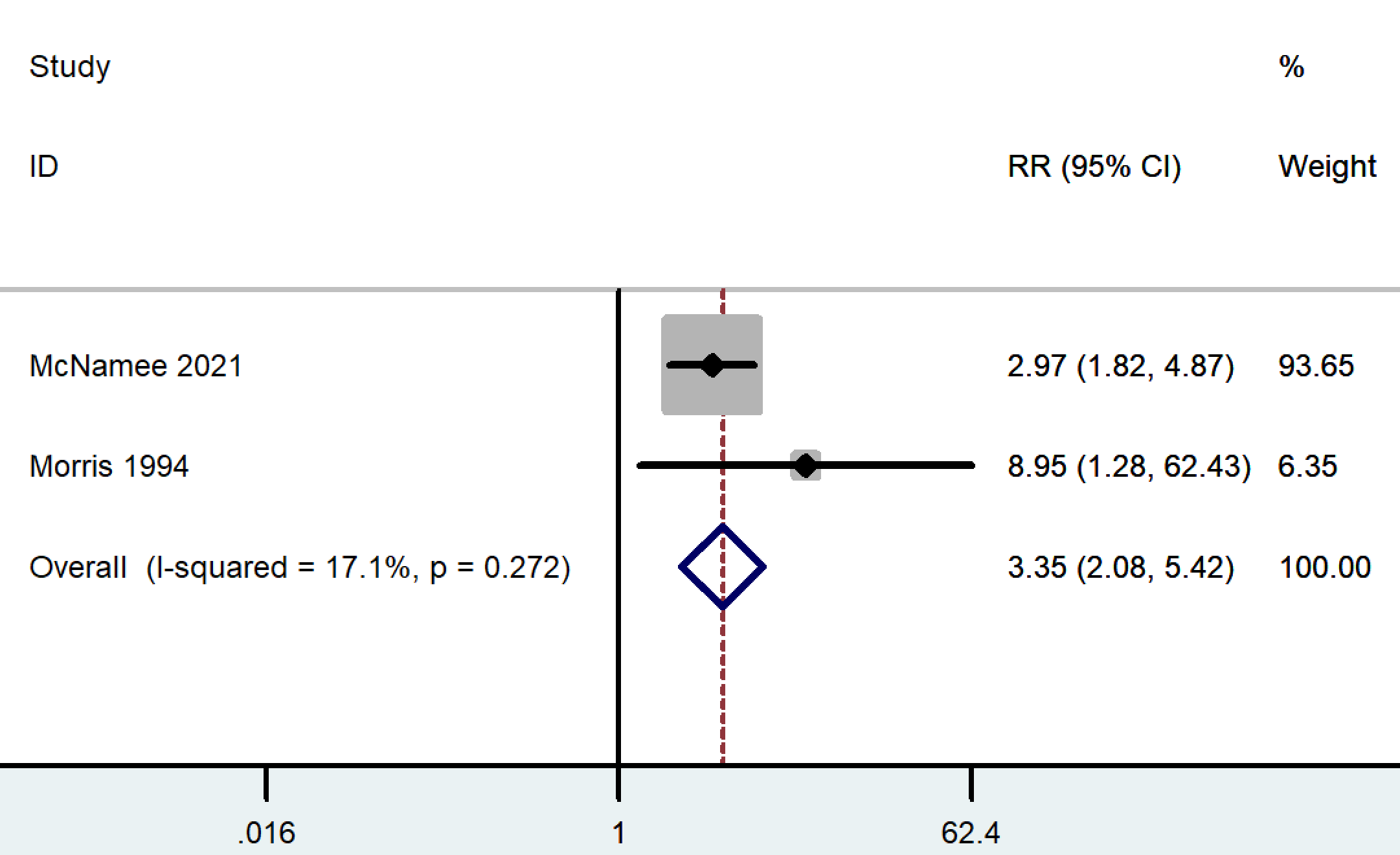


**Figure S8.** Incidence of major complications. (RR 3.35, 95% CI 2.08 to 5.42, P<0.01, I^2^ =17.1%)
