# Supplementary material for: Extracorporeal carbon dioxide removal for patients with acute respiratory failure: a systematic review and meta-analysis
Source: Ann Med. 2023 Mar 1;55(1):746–59. doi: 10.1080/07853890.2023.2172606 (PMC9980035; doi:10.1080/07853890.2023.2172606)
Supplement: Supplemental Material [file IANN_A_2172606_SM9105.docx]

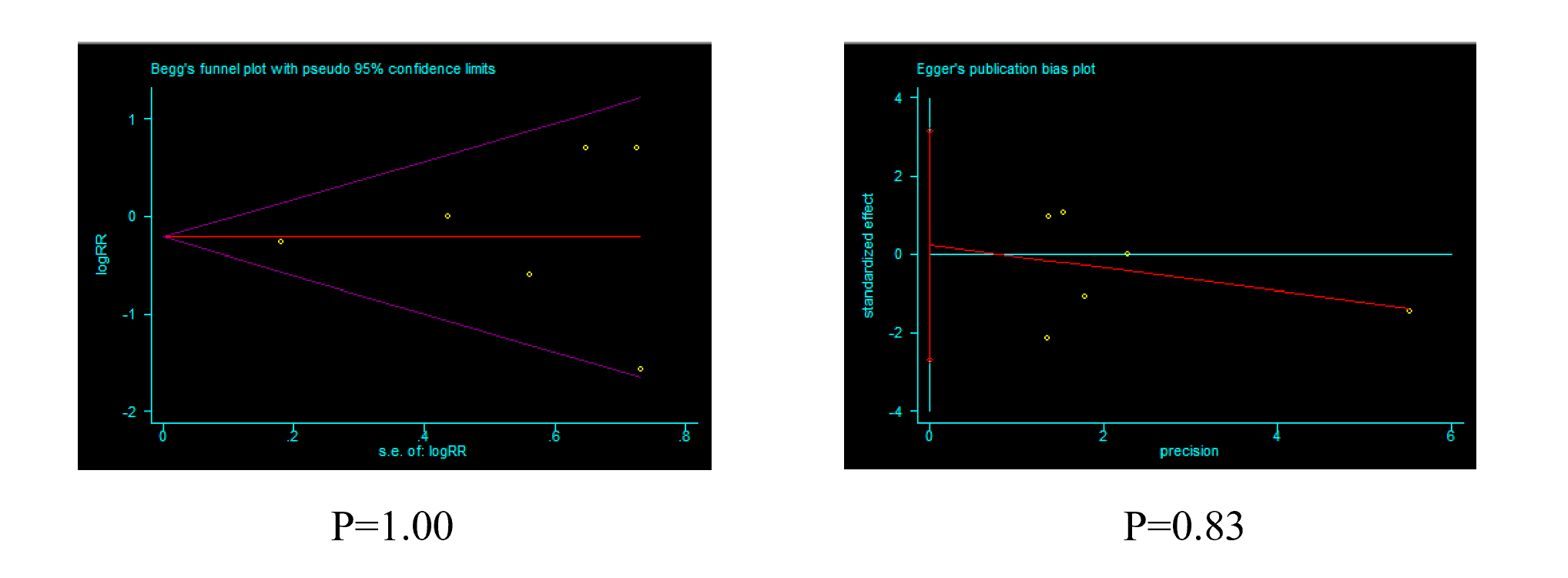


Fig.1 The Begg’s funnel plots and Egger’s test of mortality in ARF patients secondary to COPD.


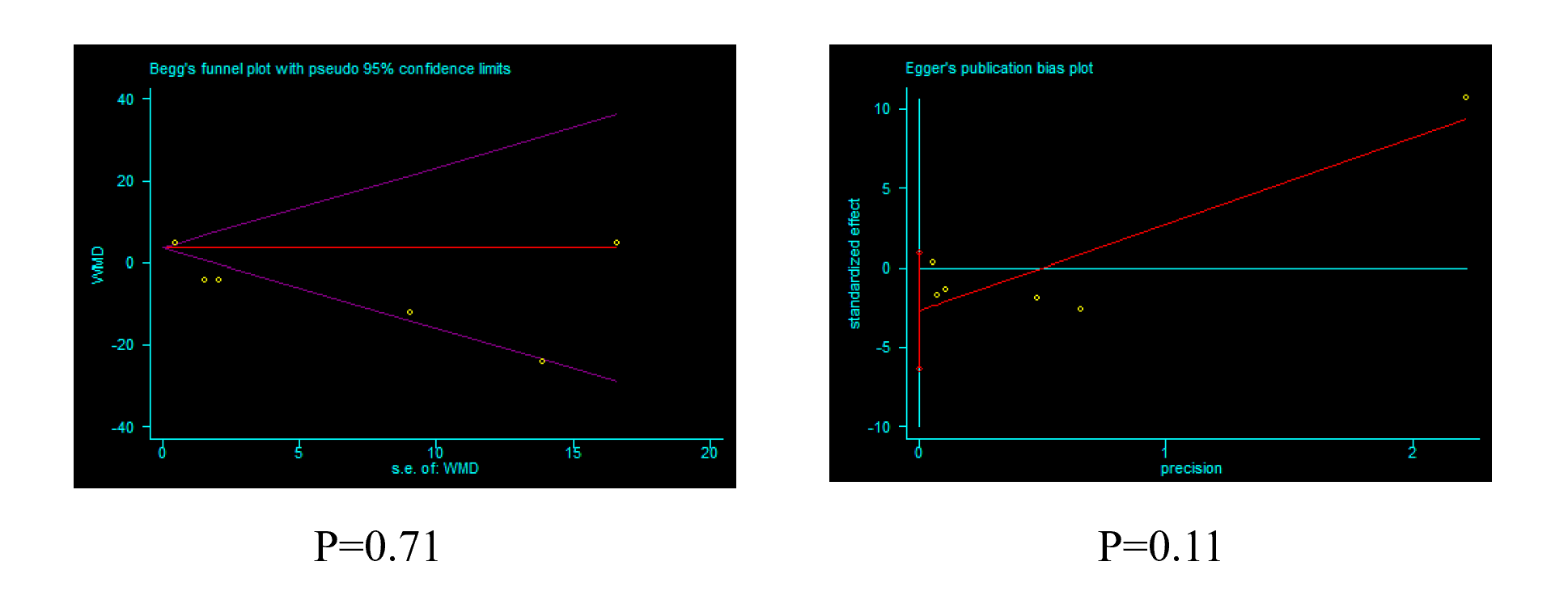


Fig.2 The Begg’s funnel plots and Egger’s test of length of ICU stay in ARF patients secondary to COPD.


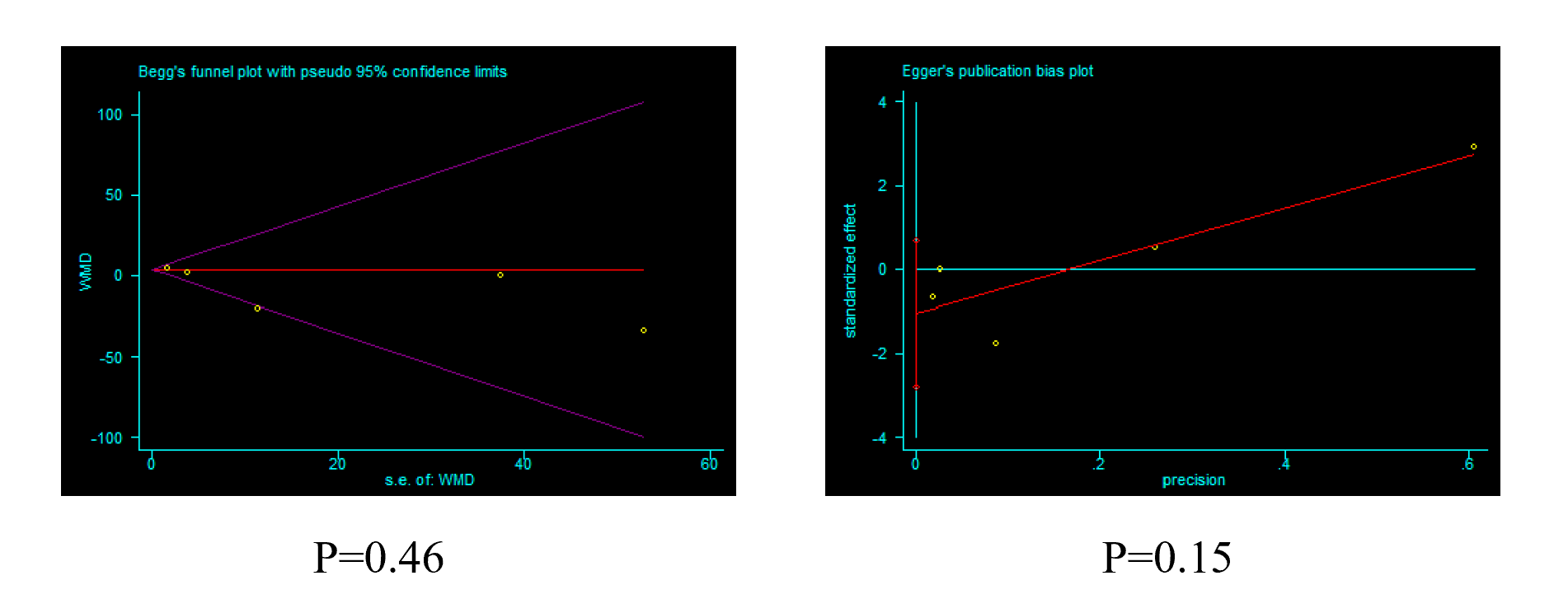


Fig.3 The Begg’s funnel plots and Egger’s test of length of hospital stay in ARF patients secondary to COPD.
